# Bruton's Tyrosine Kinase Inhibitors: Mechanisms, Efficacy, Toxicities and Applications for the Treatment of Human Diseases

**DOI:** 10.1002/mco2.71029

**Published:** 2026-10-02

**Authors:** Miao Sun, Dengju Li, Jia Wei

**Affiliations:** ^1^ Department of Hematology, Tongji Hospital, Tongji Medical College Huazhong University of Science and Technology Wuhan China; ^2^ Immunotherapy Research Center for Hematologic Diseases of Hubei Province Wuhan China

**Keywords:** autoimmune diseases, B‐cell malignancy, Bruton's tyrosine kinase (BTK) inhibitors, combination therapy, immunotherapy

## Abstract

Bruton's tyrosine kinase (BTK) is a nonreceptor tyrosine kinase that couples B‐cell receptor (BCR) signaling to broader immune‐cell activation. By integrating signals from BCR, Toll‐like receptors, chemokine receptors, Fc receptors, and BAFF receptors, BTK governs B‐cell development, immune‐cell migration, innate immunity, and inflammation. Inhibition of BTK has transformed the treatment landscape of B‐cell malignancies, while the development of covalent, noncovalent, dual‐target inhibitors, and BTK degraders has expanded therapeutic opportunities. However, the mechanisms of action, resistance patterns, and expanding therapeutic applications of BTK inhibitors (BTKis) across malignant and nonmalignant diseases have yet to be comprehensively integrated. This review outlines BTK structure and signaling pathways, BTKi classification, resistance mechanisms, and emerging therapeutic strategies with emphasis on monotherapy in B‐cell malignancies and combination strategies involving CAR‐T therapy, immune checkpoint inhibitors, CD20‐targeted antibodies, BCL2 inhibitors, and hematopoietic stem cell transplantation. Mechanistically, these combinations enhance T‐cell function, attenuate immune checkpoint signaling, and remodel the tumor microenvironment (TME). We further discuss BTKis in solid tumors, autoimmune diseases, and chronic inflammatory disorders. Collectively, this review integrates current advances in BTK‐targeted therapy and highlights opportunities for precision treatment across oncology and immune‐mediated diseases.

## Graphical abstract

BTK inhibitors suppress malignant B‐cell signaling while simultaneously reshaping immune regulation through effects on T cells and the tumor microenvironment. These mechanisms support combination immunotherapy strategies and also underlie the expanding application of BTK inhibitors in solid tumors, autoimmune and inflammatory diseases.

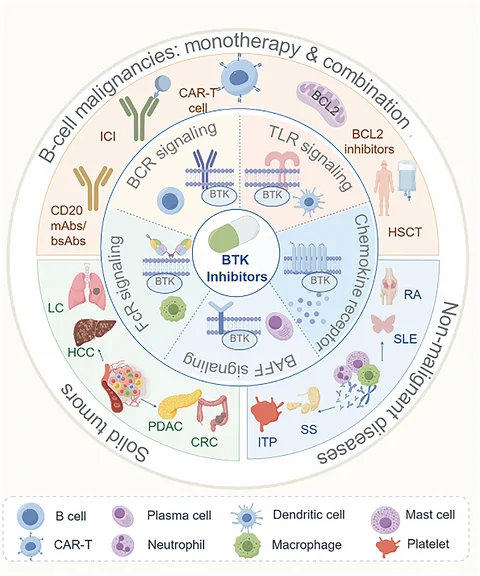

## 1 Introduction

Bruton's tyrosine kinase (BTK) was first identified in 1993 as the causative gene of X‐linked agammaglobulinemia (XLA), a disorder characterized by the absence of mature B cells and immunoglobulins [1], underscoring its essential role in B‐cell development and maturation. As a nonreceptor tyrosine kinase of the Tec family, BTK functions as a central signaling node downstream of the B‐cell receptor (BCR), coupling antigen stimulation to multiple survival pathways, including NF‐κB, PI3K/AKT, MAPK, and NFAT [2]. Dysregulated BTK activation has been established as a critical driver of several B‐cell malignancies, providing a compelling biological rationale for therapeutic targeting [3]. The advent of BTK inhibitors (BTKis) has reshaped the treatment paradigm of chronic lymphocytic leukemia (CLL) [4], mantle cell lymphoma (MCL), and Waldenström macroglobulinemia (WM), delivering durable responses even in genetically high‐risk populations, such as patients with del(17p), TP53 mutations, unmutated IGHV, or complex karyotypes [5, 6]. Nevertheless, some patients still develop resistance or experience disease progression, underscoring the limitations of monotherapy and the need for optimized treatment strategies [7].

Beyond direct cytotoxic effects on malignant B cells, accumulating evidence indicates that BTK inhibition exerts broader immunomodulatory functions. BTKis influence T‐cell activation, reduce chemokine‐mediated tumor cell homing, modulate immune checkpoint signaling, and attenuate immunosuppressive cell subsets within the tumor microenvironment (TME) [8, 9, 10]. These effects extend the conceptual framework of BTK targeting from tumor cell‐intrinsic signaling blockade to systemic immune regulation, thereby supporting rational combination strategies with chimeric antigen receptor T‐cell (CAR‐T) therapy, immune checkpoint inhibitors, CD20‐targeted antibodies, BCL2 inhibitors, and hematopoietic stem cell transplantation, which have achieved deeper responses and higher rates of minimal residual disease (MRD) clearance across multiple clinical settings.

BTK is also expressed in various innate immune cells, including macrophages, monocytes, dendritic cells, neutrophils, and mast cells, where it participates in Fc receptor (FcR), Toll‐like receptor (TLR), and B‐cell activating factor receptor (BAFF‐R) signaling [11, 12]. Dysregulated BTK activity contributes to aberrant immune activation, autoantibody production, and chronic inflammation [13]. These findings extend the therapeutic potential of BTKis to autoimmune and inflammatory diseases. Moreover, emerging evidence indicates that aberrant BTK isoforms, particularly p65BTK, are involved in the pathogenesis of several solid tumors, further broadening the scope of BTK‐targeted therapy beyond hematologic malignancies [14, 15]. Clinical and translational studies have reported encouraging efficacy and safety profiles, supporting BTK inhibition as a promising immunomodulatory strategy beyond oncology.

Accordingly, this review provides a comprehensive and updated overview of the role of BTKis in human diseases. We first discuss BTK biology and its downstream signaling pathways, followed by a summary of BTKi mechanisms of action, resistance, and emerging therapeutic strategies. We then detail their applications in hematologic malignancies, both as monotherapy and in combination with immunotherapies such as CAR‐T cells, immune checkpoint inhibitors, and bispecific antibodies, followed by their emerging roles in solid tumors. Finally, given the growing recognition of BTK inhibition as a novel anti‐inflammatory approach, we summarize recent advances in autoimmune and chronic inflammatory diseases, aiming to provide translational insights for the optimization of future BTK‐targeted therapies.

## 2 BTK Structure and Signaling Pathways

BTK acts as a central intracellular signaling hub that transduces receptor‐derived immune cues into coordinated cellular, inflammatory, and microenvironmental responses. As a key mediator of BCR, Toll‐like receptors (TLRs), chemokine receptors, Fc receptors (FcRs), and B‐cell activating factor (BAFF) receptors signaling, BTK coordinates B‐cell survival, development, differentiation, migration, innate immune activation, antibody‐mediated effector functions, and interactions with the immune microenvironment. These interconnected pathways govern immune homeostasis and drive the pathogenesis of B‐cell malignancies and immune‐mediated disorders, establishing BTK as a critical therapeutic target. Understanding its structural domains and activation mechanisms is therefore essential for interpreting the pharmacologic properties of BTK inhibitors and the molecular basis of therapeutic response and resistance.

### 2.1 Structure of BTK

BTK is a 659‐amino‐acid nonreceptor tyrosine kinase of the Tec family that is predominantly expressed in hematopoietic cells, where it plays an essential role in B‐cell development and immune regulation [1, 16]. BTK is a multidomain protein comprising a pleckstrin homology (PH) domain [17], a Tec homology (TH) domain, a Src homology 3 (SH3) domain, a Src homology 2 (SH2) domain, and a kinase domain (SH1) (Figure 1A) [18, 19]. Its activity is triggered by phosphorylation at Y551 and Y223 [20, 21]. Upon activation, BTK binds ATP and substrates through its kinase domain, leading to substrate phosphorylation and propagation of downstream signaling cascades [22].

**FIGURE 1:**
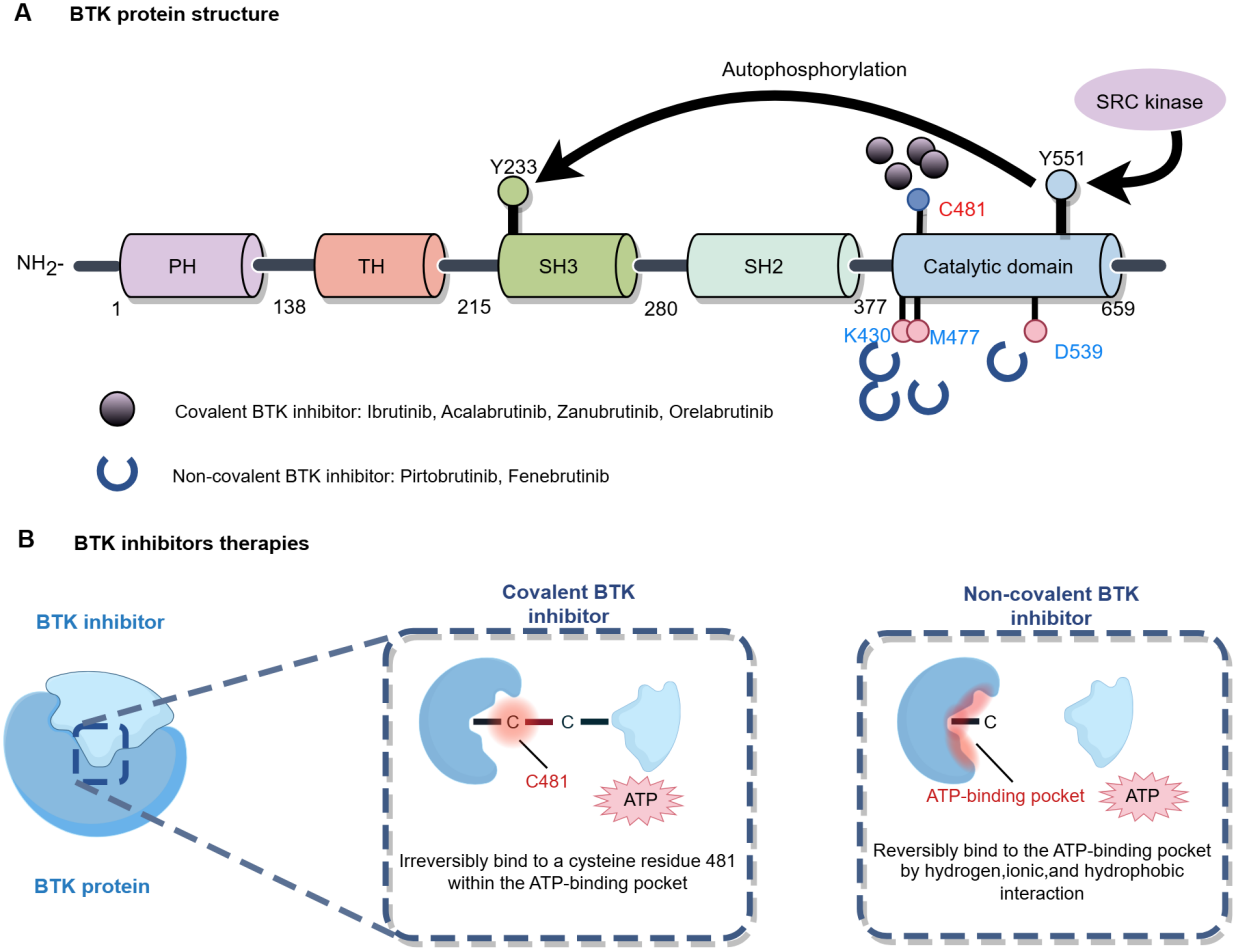
Structure of BTK and BTK inhibitor therapies. (A) PH, pleckstrin homology domain, which binds phospholipids to recruit BTK from the cytosol to the plasma membrane; TH, Tec homology domain, required for BTK stability; SH3 and SH2 domains, which mediate protein–protein interactions and contain the phosphorylation site Y233. The catalytic domain contains the phosphorylation site Y551, an ATP‐binding pocket, and C481, a critical covalent inhibitor binding site. Approved BTK inhibitors primarily target the catalytic domain: covalent inhibitors bind to C481, whereas noncovalent inhibitors bind to K430, M477, D539, and other residues. BTK is phosphorylated at Y551 by SRC family kinases, leading to autophosphorylation at Y223 in the SH3 domain. Phosphorylation at Y223 stabilizes the active conformation and fully activates BTK kinase activity. (B) Covalent BTK inhibitors irreversibly bind to C481; noncovalent BTK inhibitors occupy the ATP‐binding pocket.

### 2.2 Role of BTK in BCR Signaling During B‐Cell Development and Differentiation

Among BTK‐dependent pathways, BCR signaling is the best‐characterized mechanism linking receptor engagement to B‐cell fate decisions. BCR signaling operates through both tonic and antigen‐induced modes to sustain B‐cell homeostasis, proliferation, survival, and differentiation [23]. In antigen‐induced BCR signaling, BTK functions downstream of LYN/SYK‐ and PI3K‐dependent proximal events, coupling membrane recruitment and phosphorylation‐dependent activation to PLCγ2‐mediated downstream signaling [24]. Structurally, the BCR complex consists of membrane‐bound immunoglobulin associated with the Igα/Igβ heterodimer, also known as CD79a/CD79b [3]. Upon antigen engagement, the Src family kinase LYN phosphorylates the immunoreceptor tyrosine‐based activation motifs (ITAMs) on the cytoplasmic tails of CD79a/b, recruiting and activating SYK [3, 13, 23]. In parallel, PI3K generates the second messenger phosphatidylinositol‐3,4,5‐trisphosphate (PIP3), which recruits BTK to the plasma membrane via its PH domain. BTK is subsequently activated through dual phosphorylation at Y551 and Y223 [13, 17] and then phosphorylates PLCγ2 at Y753 and Y759, generating two key second messengers: inositol trisphosphate (IP3) and diacylglycerol (DAG) [25]. IP3 regulates intracellular Ca^2^^+^ levels and activates the transcription factor nuclear factor of activated T cells (NFAT) via calmodulin, whereas DAG promotes assembly of the CARD11‐BCL10‐MALT1 scaffold complex to initiate the NF‐κB signaling cascade, thereby inducing the expression of Bcl‐xL and cyclins [26]. DAG also activates the RAS‐RAF‐MEK‐ERK pathway, enhancing transcription of cell cycle regulators and antiapoptotic proteins [2, 15, 26, 27] (Figure 2). These cascades collectively govern B‐cell survival, proliferation, apoptosis, differentiation, and antibody production. In the absence of BTK, reduced Bcl‐xL induction via BCR signaling leads to increased B‐cell apoptosis.

**FIGURE 2:**
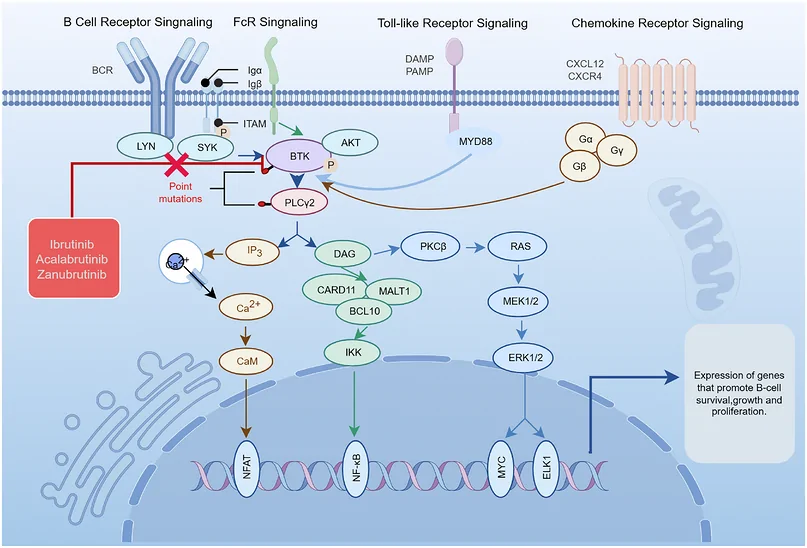
BTK regulates multiple receptor signaling pathways, including B‐cell receptor (BCR), Toll‐like receptor (TLR), chemokine receptor, and FcR pathways. BCR signaling drives B‐cell proliferation and survival. BTK also mediates antigen‐independent TLR and chemokine receptor signaling, regulating B‐cell adhesion, migration, and interactions within the tumor microenvironment (TME). Mutations in BTK C481 or in downstream PLCγ2 disrupt covalent BTK inhibitor (ibrutinib, acalabrutinib, zanubrutinib) binding, restoring BTK catalytic activity.

BTK plays a critical role in early B‐cell development. In the bone marrow, successful rearrangement of the immunoglobulin heavy chain triggers pre‐BCR signaling [3, 28, 29]. Together with BLNK (also known as SLP65) and PLCγ2, BTK mediates downstream signaling required for progression to the immature B‐cell stage [3, 30]. In XLA, mutations in the BTK gene cause developmental arrest at the pro‐B‐cell stage, underscoring its nonredundant function [29]. Notably, BTK also cooperates with BLNK to regulate IL‐7‐dependent proliferation and pre‐B‐cell survival by inhibiting JAK‐STAT5 activation, suggesting a tumor‐suppressive role during early leukemogenesis [31, 32, 33].

BTK is essential for peripheral B‐cell maturation, particularly in supporting T2 cell survival and phenotypic transition [34, 35, 36, 37]. Its dysregulation contributes to constitutive BCR signaling in B‐cell malignancies, promoting aberrant survival and proliferation [38].

### 2.3 Role of BTK in Regulating TLR Signaling and Immune Modulation

BTK modulates innate immune signaling by regulating TLR pathways, particularly those mediated by TLR9 and TLR4 [39]. TLRs are key sensors that bridge innate and adaptive immunity [40, 41]. Their extracellular domains recognize pathogen‐associated or damage‐associated molecular patterns (PAMPs/DAMPs) and initiate signaling via intracellular Toll/interleukin‐1 receptor (TIR) domains. Upon activation, most TLRs recruit the adaptor myeloid differentiation primary response protein 88 (MyD88) [40, 42, 43], which in turn activates interleukin‐1 receptor‐associated kinase 1 (IRAK1), either directly or in conjunction with the TIR domain‐containing adaptor protein (TIRAP; also known as MAL). BTK interacts with TLR TIR domains, MyD88, IRAK1, and TIRAP to promote activation of the transcription factors NF‐κB, AP‐1, and IRF3 [44, 45, 46]. These events drive B‐cell activation and proliferation, antibody production with class‐switch recombination, and proinflammatory cytokine secretion [47, 48, 49] (Figure 2).

Beyond these proximal signaling roles, BTK contributes to immune regulation by fostering the differentiation of IL‐10‐producing regulatory B cells, thereby maintaining immune tolerance [39], and by restraining inflammatory T‐cell subsets (Th1 and Th17) in autoimmune settings to promote disease resolution [47, 50, 51].

Dysregulated TLR signaling can also contribute to lymphomagenesis. In activated B‐cell‐like diffuse large B‐cell lymphoma, approximately 39% of cases harbor MyD88 mutations that drive constitutive NF‐κB activation [52, 53, 54]. The resulting inflammatory microenvironment exacerbates DNA damage and genomic instability [47, 48], underscoring the pathogenic relevance of TLR‐BTK crosstalk.

### 2.4 Role of BTK in Chemokine Receptor‐Mediated Migration

Biochemical and in vitro adhesion/migration assays demonstrate that BTK plays a key role in chemokine receptor signaling, regulating B‐cell migration and homing [42, 55]. It acts downstream of chemokine receptors such as CXCR4 and CXCR5, directing B‐cell trafficking to lymphoid niches [13, 56, 57]. Upon CXCL12 binding to CXCR4, BTK is activated via interactions with Gα and Gβγ subunits [58, 59]. Activated BTK phosphorylates PLCγ2 and regulates cytoskeletal rearrangement, adhesion molecule expression, and integrin activation, thereby promoting B‐cell homing and retention in the bone marrow and lymph nodes [55] (Figure 2). In malignant B cells, BTK‐mediated chemokine signaling supports trafficking to protective niches such as the bone marrow and lymphoid tissues, contributing to microenvironment‐mediated drug resistance.

### 2.5 Role of BTK in FcR Signaling Pathway

Beyond B cells, BTK plays a critical role in Fc receptor (FcγR and FcεR) signaling in myeloid cells, including monocytes, macrophages, neutrophils, mast cells, and basophils [60] (Figure 2). Fcγ receptors, members of the immunoglobulin superfamily, comprise both activating and inhibitory subtypes. Immune complex engagement of FcγR activates SYK, which subsequently recruits and activates BTK, leading to downstream PLCγ2/Ca^2^^+^ signaling as well as activation of NF‐κB and MAPK pathways [61]. These signals promote proinflammatory cytokine production (e.g., TNF‐α, IL‐6, and IL‐1β), oxidative burst, degranulation, and antibody‐dependent cellular phagocytosis (ADCP) [60, 62]. Persistent immune complexes in circulation or tissues result in sustained pathway activation, driving immune complex‐mediated inflammation, tissue injury, and alterations in the TME [63].

In the tumor setting, FcR‐BTK signaling primarily regulates myeloid cell function, particularly that of TAMs [64]. BTK activation promotes macrophage polarization toward an immunosuppressive phenotype and enhances IL‐10 and TGF‐β secretion, thereby suppressing antitumor T‐cell responses [60]. This pathway contributes to tumor growth, angiogenesis, and metastasis. Pathologically, excessive FcR‐BTK activation also drives immune complex‐mediated inflammatory diseases [65]. It promotes synovial inflammation and bone destruction in rheumatoid arthritis, enhances immune complex‐driven interferon responses and plasmacytoid dendritic cell activation in systemic lupus erythematosus, and mediates macrophage‐dependent platelet clearance in immune thrombocytopenia [66, 67]. In allergic diseases, FcεR‐dependent BTK activation in mast cells and basophils further induces histamine release and other inflammatory mediators [60, 64].

### 2.6 Role of BTK in B‐Cell Activating Factor (BAFF) Receptor Signaling Pathway

BAFF, a member of the tumor necrosis factor (TNF) ligand family, cooperates with BCR signaling to regulate the survival and homeostatic proliferation of mature B cells [28]. BAFF primarily activates the noncanonical NF‐κB pathway via IKKα and also promotes BTK‐dependent survival signaling through the PI3K/AKT and NF‐κB pathways, leading to increased expression of anti‐apoptotic proteins such as BCL‐2 and MCL‐1 [26, 68, 69]. Furthermore, during B‐cell activation, BAFF supports immunoglobulin class switching and differentiation into plasma cells [70].

Dysregulated BAFF signaling disrupts B‐cell tolerance and selection. In mouse models, BAFF overexpression promotes the survival of autoreactive B cells and leads to systemic autoimmune phenotypes resembling systemic lupus erythematosus and Sjögren's syndrome [71]. Excess BAFF can also rescue anergic autoreactive B cells, enhance follicular dendritic cell network formation, and support germinal center development, affinity maturation, and sustained autoantibody production [72, 73].

In summary, BTK integrates BCR, TLR, chemokine receptor, FcR, and BAFF signaling to orchestrate B‐cell development and malignancy, establishing it as a rational therapeutic target.

## 3 BTK Inhibitors: Mechanisms, Resistance, and Emerging Therapeutic Strategies

The central role of BTK in BCR signaling and its selective expression in B cells establish it as an attractive therapeutic target [74, 75]. BTK inhibitors disrupt downstream signaling by blocking BTK autophosphorylation, thereby inducing apoptosis and inhibiting proliferation through the downregulation of Bcl‐xL and cyclin D2 [26, 76]. Clinically, these agents have demonstrated significant efficacy in CLL, MCL, FL, WM, and other B‐cell malignancies, increasingly replacing traditional chemotherapy [42] (Figure 3).

**FIGURE 3:**
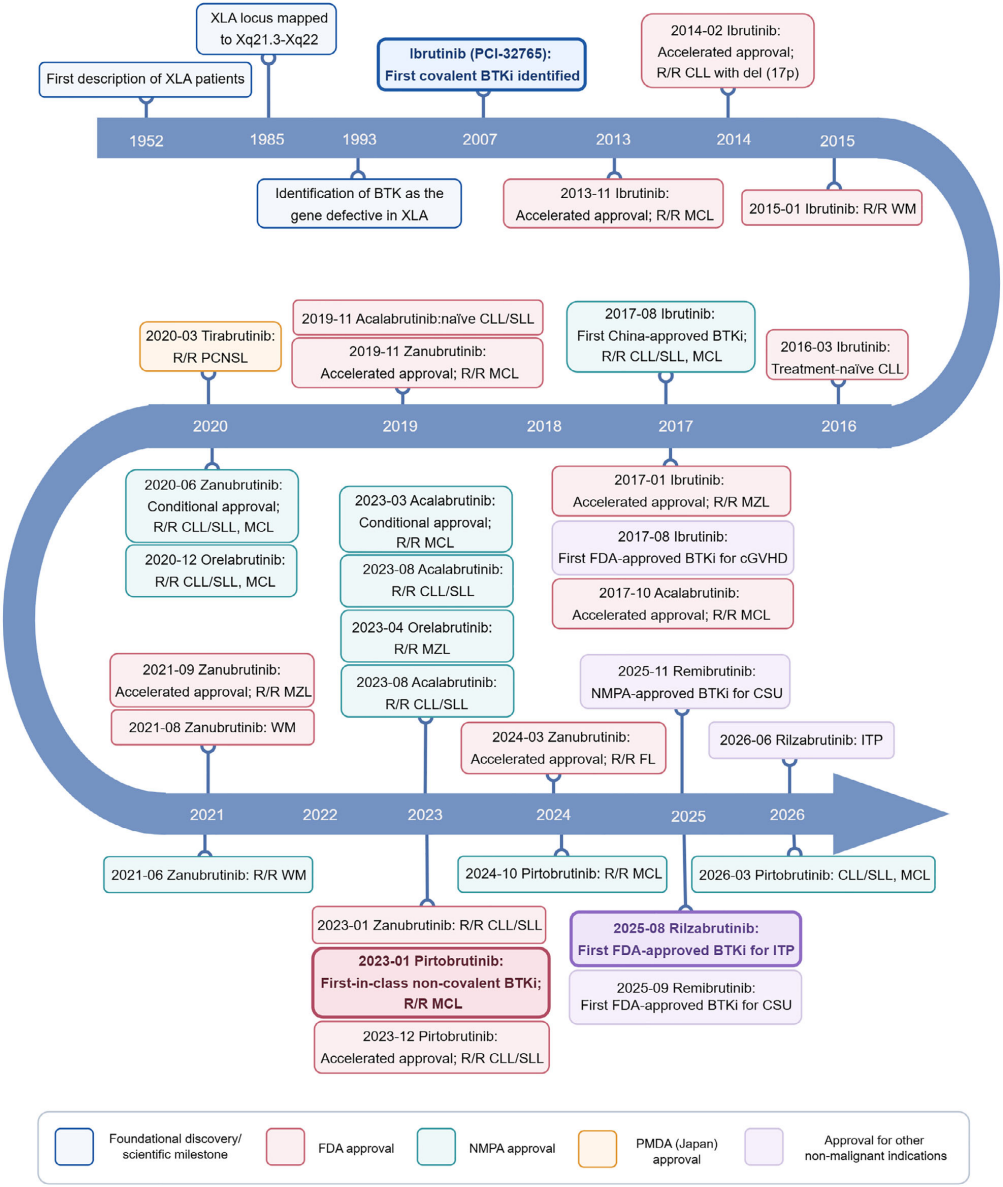
Key milestones in BTK target discovery and inhibitor development, with FDA‐ and NMPA‐approved indications. BTK‐targeted therapy development began with identification of the BTK gene in 1993 following discovery of X‐linked agammaglobulinemia (XLA). Ibrutinib, the first‐generation covalent BTK inhibitor, received FDA approval in 2013 for relapsed/refractory mantle cell lymphoma (R/R MCL), marking a 20‐year transition from target discovery to clinical application. Subsequently, covalent and noncovalent BTK inhibitors have been approved by the FDA and NMPA for hematologic malignancies and immune‐mediated diseases. These milestones highlight the expansion of BTK‐targeted therapy from B‐cell malignancies to broader indications, including chronic graft‐versus‐host disease, immune thrombocytopenia, and chronic spontaneous urticaria. BTK, Bruton's tyrosine kinase; BTKi, BTK inhibitor; FDA, U.S. Food and Drug Administration; NMPA, National Medical Products Administration; PMDA, Pharmaceuticals and Medical Devices Agency; XLA, X‐linked agammaglobulinemia; R/R, relapsed/refractory; CLL/SLL, chronic lymphocytic leukemia/small lymphocytic lymphoma; MCL, mantle cell lymphoma; WM, Waldenström macroglobulinemia; MZL, marginal zone lymphoma; FL, follicular lymphoma; PCNSL, primary central nervous system lymphoma; cGVHD, chronic graft‐versus‐host disease; ITP, immune thrombocytopenia; CSU, chronic spontaneous urticaria.

### 3.1 Action and Classification of BTK Inhibitors

BTK inhibitors target the ATP‐binding site via covalent or noncovalent interactions (Figure 1B) [77]. Covalent inhibitors bind irreversibly to C481, blocking Y223 phosphorylation while sparing Y551 [77, 78]. In contrast, noncovalent inhibitors act independently of C481, forming hydrogen bonds and hydrophobic interactions within the ATP pocket to inhibit both Y223 and Y551 phosphorylation [22, 42, 79, 80]. Both classes suppress downstream signaling (e.g., PLCγ2, ERK, NF‐κB), reducing B‐cell proliferation and inducing apoptosis. Ibrutinib, a first‐generation covalent inhibitor, improved outcomes in B‐cell malignancies but is limited by off‐target toxicity and resistance driven by C481 mutations [81, 82, 83]. Second‐generation covalent inhibitors (acalabrutinib, zanubrutinib, and orelabrutinib) offer improved selectivity for C481 and reduced off‐target kinase inhibition (Table 1) [6, 84, 85, 86, 87], yet resistance mediated by C481S, T474, and downstream mutations persists [81]. Third‐generation noncovalent inhibitors (pirtobrutinib, nemtabrutinib, and vecabrutinib) overcome C481‐related resistance (Tables 1 and 2) [88, 89, 90]. Pirtobrutinib, the only approved agent in this class, forms a three‐dimensional hydrogen‐bond network within the ATP‐binding site, retains activity against wild‐type and mutant BTK, and exhibits >300‐fold selectivity for BTK over 98% of other kinases [88, 90]. It also achieves approximately 90‐fold higher peak plasma concentrations and a 2.5‐fold longer half‐life than ibrutinib [91].

**TABLE 1:** Summary of approved BTK inhibitors and resistance mechanisms.

BTK inhibitor	Type of inhibitor	Approved indications	Year of approval	Resistance mechanisms	Comments	Common Nonhematologic AEs (grade ≥3)	Refs.
Ibrutinib	Covalent	CLL/SLL, MCL, WM, cGVHD, MZL (FDA) CLL/SLL, MCL, WM (NMPA)	2013 (FDA) 2017 (NMPA)	C481S mutation; PLCγ2 downstream mutation.	First‐in‐class BTK inhibitor	Hypertension (approx. 11%–23%); Atrial fibrillation/flutter (approx. 3%–6%); Pneumonia/Infection (approx. 10%–15%); Diarrhea (approx. 4%)	[6, 97, 98, 99]
Acalabrutinib	Covalent	CLL/SLL, MCL(FDA) CLL/SLL, MZL (NMPA)	2017 (FDA) 2023 (NMPA)	C481S mutation; PLCγ2 downstream mutation.	No ITK or EGFR inhibition	Hypertension (approx. 4%–9%); Infection (approx. 5%–10%); Atrial fibrillation (approx. 4.5%)	[100, 101, 102, 103]
Zanubrutinib	Covalent	CLL/SLL, MZL, MCL, FL, WM (FDA) CLL/SLL, MCL, WM, FL(NMPA)	2019 (FDA) 2020 (NMPA)	C481S and L528W mutations; PLCγ2 downstream mutation.	No ITK inhibition Dose retains TEC inhibition	Hypertension (approx. 3%–15%); Pneumonia (approx. 10%); Second primary malignancies (approx. 3%–5%)	[6, 104, 105, 106]
Tirabrutinib	Covalent	PCNSL, WM (PMDA)	2020 (PMDA) Approved in Japan only	C481S mutation; PLCγ2 downstream mutation.	Approved in Japan only	Skin rash (approx. 2%‐6%), pneumonia (approx. 6%), aspartate aminotransferase increased (approx. 4%–6%)	[107, 108]
Orelabrutinib	Covalent	CLL/SLL, MCL (NMPA)	2020 (NMPA) Approved in China only	C481S mutation; PLCγ2 downstream mutation.	Approved in China only, and higher synergy with R‐CHOP than ibrutinib	Infection (approx. 5%–8%), Skin rash/hypersensitivity (low incidence)	[109, 110]
Pirtobrutinib	Noncovalent	CLL/SLL, MCL (FDA) MCL, CLL/SLL (NMPA)	2023 (FDA) 2024 (NMPA)	Overcomes C481X mutation‐mediated resistance	Only FDA‐approved noncovalent BTK inhibitor	Infection (approx. 10%–15%), hypertension (approx. 2%), atrial fibrillation/flutter (<1%), second primary malignancies (approx. 3%–5%).	[103, 111, 112]
Rilzabrutinib	Covalent	ITP (FDA)	2025 (FDA)	N/A	The first BTK inhibitor for immune thrombocytopenia	Diarrhea (<2%), Nausea/Vomiting (<2%).	[113, 114]
Remibrutinib	Covalent	CSU (FDA, NMPA)	2025 (FDA, NMPA)	N/A	The first BTK inhibitor for CSU	Elevated ALT/AST (<2%); infection (approx. 1%–3%); petechiae (approx. 3.8%).	[115]

*Note*: Data sources: NMPA, FDA, and Nature Reviews Drug Discovery.

Abbreviations: AE, adverse event; ALT, alanine aminotransferase; AST, aspartate aminotransferase; BTK, Bruton's tyrosine kinase; cGVHD, chronic graft‐versus‐host disease; CLL, chronic lymphocytic leukemia; CSU, chronic spontaneous urticaria; EGFR, epidermal growth factor receptor; FL, follicular lymphoma; ITK, interleukin‐2–inducible T‐cell kinase; ITP, immune thrombocytopenia; MCL, mantle cell lymphoma; MZL, marginal zone lymphoma; N/A, not applicable; NMPA, National Medical Products Administration; PCNSL, primary central nervous system lymphoma; PMDA, Pharmaceuticals and Medical Devices Agency; SLL, small lymphocytic lymphoma; TEC, Tec protein tyrosine kinase; WM, Waldenström macroglobulinemia. FDA, U.S. Food and Drug Administration.

**TABLE 2:** Summary of BTK inhibitors and BTK degraders in ongoing clinical trials.

Category	BTK inhibitor	Indications	Clinical phase	Company	Comments	NCT
Covalent	Spebrutinib CC‐292	CLL, DLBCL	II	Avila/Celgene	High BTK selectivity, but limited clinical progress	NCT02031419 NCT01766583 NCT01351935
	TL‐895	B‐NHL, MCL, DLBCL	I/II	Merck	Enhanced compatibility with ADCC‐based therapeutic antibodies; potential advantage over ibrutinib in combination settings	NCT04419623 NCT02825836
	DTRMWXHS‐12	R/R CLL, R/R NHL, MCL	I	DTRM Biopharma	Improved pharmacokinetic profile; preliminary clinical activity demonstrated in patients	NCT03836768 NCT02891590 NCT02900716
	Rilzabrutinib SAR444671	ITP, CSU, wAIHA, asthma	II. III	Sanofi	Reversible covalent BTK inhibitor enabling durable target engagement with reduced off‐target toxicity	NCT04562766 NCT05107115 NCT05002777 NCT05104892
	Orelabrutinib	MCL, MZL, PCNSL, SCNSL, ITP, MS, SLE	II, III	BeiGene	Minimal off‐target kinase activity allows sustained BTK occupancy and an improved safety profile	NCT07299019 NCT07185373 NCT07372365 NCT07410520 NCT07400250
	Evobrutinib M52951	RA, MS	II	Merck	Moderate blood–brain barrier penetration enables modulation of B‐cell and innate immune‐driven inflammation	NCT03233230 NCT04032158
	Branebrutinib BMS‐986195	SLE, RA, atopic dermatitis	II	Bristol Myers Squibb	Highly potent irreversible BTK inhibitor with rapid and sustained target occupancy and strong selectivity	NCT05014438 NCT04186871
	CC‐292	R/R B‐cell malignancies, RA	I, II	Celgene	Did not achieve significant clinical improvement in arthritis symptoms but demonstrated reduction in inflammatory biomarkers	NCT01766583 NCT01975610
	Remibrutinib	CSU, MS	II, III	Novartis	Designed to achieve high target occupancy while minimizing off‐target effects, particularly hepatotoxicity	NCT07225504 NCT07222956 NCT07408219
	Tolebrutinib SAR442168	MS	III	Sanofi	Brain‐penetrant BTK inhibitor with high blood–brain barrier permeability	NCT03996291 NCT06372145
Noncovalent	Nemtabrutinib ARQ‐531	CLL/SLL, MCL, FL, WM	II	Merck	Overcomes C481X and PLCγ2 mutation‐mediated resistance	NCT06793371 NCT03162536 NCT05624554
	Vecabrutinib SNS‐062	CLL/SLL, MCL	I/II	Sunesis	Development discontinued due to insufficient clinical efficacy	NCT03037645
	Fenebrutinib GDC‐0853	SLE, MS, CSU	II, III	Roche	Effective central nervous system penetration enables modulation of peripheral B cells and microglia‐driven inflammation	NCT02908100 NCT07161258 NCT04544449 NCT03137069
PROTAC degrader	NX‐2127	R/R DLBCL, WM, MCL, CLL/SLL	Ib	Nurix	Induces selective degradation of BTK, IKZF1, and IKZF3; overcomes resistance from both kinase‐impaired and kinase‐proficient mutations	NCT04830137
	BGB‐16673	R/R B‐cell malignancies	III	BeiGene	Broad‐spectrum BTK degrader targeting both covalent and noncovalent resistance mutations, including C481 variants; early clinical data demonstrate promising efficacy	NCT05006716 NCT05294731
	NX‐5948	R/R B‐cell malignancies	Ia	Nurix	Demonstrates high early response rates with effective blood–brain barrier penetration and favorable tolerability	NCT05131022
	HSK29116	R/R B‐cell malignancies	I	Haisco	Exhibits dual antitumor activity through BTK kinase inhibition and proteasomal degradation	NCT04861779
	AC676	B‐cell malignancies	I	Accutar Bio	Overcomes PLCγ2‐mediated resistance by disrupting BTK scaffolding functions	NCT05780034
	ABBV‐101	R/R B‐cell malignancies	I	AbbVie	Highly selective BTK inhibitor with potent activity against both wild‐type and treatment‐resistant mutant forms	NCT05753501
Dual covalent and noncovalent	Rocbrutinib LP‐168	R/R DLBCL, MCL, MZL, CLL/SLL, non‐GCB DLBCL	III	Newave	Dual covalent and noncovalent binding enables inhibition of both wild‐type and mutant BTK, including C481 and T474 variants	NCT05716087 NCT06251180 NCT07377578 NCT07189065
LYN/BTK dual inhibitor	DZD8586	R/R CLL/SLL, R/R B‐NHL	II	Dizal	First dual LYN/BTK inhibitor targeting both BTK‐dependent and BTK‐independent BCR signaling; overcomes degrader resistance and demonstrates high selectivity among TEC kinases	NCT06539182 NCT05824585

Abbreviations: B‐NHL, B‐cell non‐Hodgkin lymphoma; BTK, Bruton's tyrosine kinase; CLL, chronic lymphocytic leukemia; CSU, chronic spontaneous urticaria; DLBCL, diffuse large B‐cell lymphoma; FL, follicular lymphoma; ITK, interleukin‐2‐inducible T‐cell kinase; ITP, immune thrombocytopenia; MCL, mantle cell lymphoma; MS, multiple sclerosis; MZL, marginal zone lymphoma; PCNSL, primary central nervous system lymphoma; PLCγ2, phospholipase C gamma 2; R/R, relapsed or refractory; RA, rheumatoid arthritis; SCNSL, secondary central nervous system lymphoma; SLE, systemic lupus erythematosus; wAIHA, warm autoimmune hemolytic anemia; WM, Waldenström macroglobulinemia.

*Data sources*: ClinicalTrials.gov (clinicaltrials.gov) and PubMed.

### 3.2 Resistance Mechanisms to BTK Inhibitors

Resistance to covalent BTK inhibitors such as ibrutinib frequently arises from point mutations at C481, most notably C481S. These mutations disrupt covalent drug binding while preserving BTK kinase activity and maintaining BCR signaling [79, 92, 93]. Noncovalent inhibitors like pirtobrutinib were developed to overcome C481‐mediated resistance; however, recent genomic analyses indicate that resistance to pirtobrutinib is characterized by dynamic clonal evolution rather than a single dominant mutation. Longitudinal sequencing has revealed heterogeneous non‐C481 BTK variants accompanied by clonal competition and temporal shifts in mutational dominance. Notably, kinase‐impaired BTK mutants capable of sustaining downstream signaling through scaffold functions have been identified, highlighting a catalytic activity‐independent resistance mechanism. Additionally, gain‐of‐function mutations in phospholipase C gamma 2 (PLCG2) often co‐occur with BTK mutations [94, 95]. These PLCG2 alterations enable NF‐κB activation independent of BTK, representing a bypass resistance mechanism particularly prevalent in patients with CLL or Waldenström macroglobulinemia who develop ibrutinib resistance [96].

In addition to C481 mutations, substitutions at the T474 gatekeeper residue, such as T474I and T474S, contribute significantly to resistance [116]. These mutations alter the conformation and polarity of the kinase ATP‐binding pocket, impairing the binding of both ATP and BTK inhibitors [79, 117, 118]. Furthermore, mutations in the SH2 domain, such as T316A, disrupt interactions with phosphotyrosine‐containing adaptor proteins like BLNK, interfering with BTK‐mediated signaling complex assembly and further promoting resistance [117, 119].

Functionally, BTK resistance mutations can be broadly classified into two categories. Kinase‐proficient mutations, such as C481S, T474I, and T474F, retain enzymatic activity and sustain BCR signaling through intact BTK [7]. Kinase‐impaired mutations, including M437R, L528W, and C481R, compensate for loss of BTK activity by activating downstream signaling components, thereby maintaining BCR signaling via alternative pathways [7, 83].

Notably, clinical resistance frequently occurs in the absence of detectable BTK or PLCG2 mutations, suggesting activation of bypass signaling pathways [7]. Following BTK inhibition, malignant cells may maintain survival and proliferation through compensatory networks. Persistent activation of the PI3K/AKT/mTOR pathway supports metabolic reprogramming, protein synthesis, and anti‐apoptotic programs and, together with integrin‐mediated adhesion, has been shown to confer TME‐driven ibrutinib resistance in MCL [120]. In parallel, activation of canonical and noncanonical NF‐κB signaling restores survival by upregulating antiapoptotic proteins such as BCL‐XL and MCL‐1 and enhancing inflammatory and microenvironmental interactions [121]. In MCL, MALT1 overexpression promotes NF‐κB activation and tumor cell persistence [122], while mutations in CARD11 or TNFAIP3 lead to constitutive NF‐κB activation and contribute to ibrutinib resistance in ABC‐type diffuse large B‐cell lymphoma [123].

Chromosomal abnormalities represent an additional mechanism of resistance by enabling the selection of aggressive subclones under therapeutic pressure [124]. In CLL, gain of chromosome 2p and deletion of 8p21 have been associated with treatment resistance [125]. Similarly, deletions of 6q and 8p in Waldenström macroglobulinemia (WM) correlate with reduced sensitivity to ibrutinib. Of particular interest, amplification of the nuclear export protein XPO1, located on chromosome 2p, leads to dysregulated nuclear trafficking of key regulators including p53, FOXO, NF‐κB, and RB1, thereby promoting cell survival and cell‐cycle dysregulation [126, 127]. These findings highlight the critical role of genomic evolution and clonal selection in adaptive resistance to BTK inhibition.

### 3.3 Moving Beyond Traditional BTK Inhibitors

Non‐C481 mutations in the BTK kinase domain can confer resistance to both covalent and noncovalent BTK inhibitors, including pirtobrutinib [89, 128]. These findings highlight the limitations of ATP‐competitive inhibition in the setting of structurally diverse resistance variants and have prompted the development of next‐generation agents designed to achieve broader target engagement and more durable pathway suppression.

To address this, novel BTK inhibitors are being developed with improved safety and resistance profiles. LP‐168 is a next‐generation BTK inhibitor featuring dual covalent and noncovalent binding that retains activity against both wild‐type and C481‐mutant BTK [129]. Early trials in relapsed/refractory (R/R) B‐cell malignancies have shown promising safety and efficacy, particularly in high‐risk CLL and B‐NHL [130, 131]. In 2024, LP‐168 received Breakthrough Therapy Designation from China's NMPA for R/R non‐GCB DLBCL after ≥2 prior lines of therapy [132]. These data support dual‐binding strategies as a means of maintaining target inhibition across multiple resistance genotypes.

Birelentinib (DZD8586) is the first noncovalent dual BTK/LYN inhibitor with full blood–brain barrier penetration. It blocks both BTK‐dependent and BTK‐independent BCR signaling, thereby addressing bypass signaling pathways that may emerge under selective BTK inhibition, while also overcoming degrader‐associated mutations and sparing other TEC family kinases [133]. It has received FDA Fast Track designation for R/R CLL/SLL and is currently under evaluation in a phase 2 trial (TAI‐SHAN8, NCT06539182) and a phase 1 trial (TAI‐SHAN5, NCT05824585) in high‐risk CLL/SLL and B‐NHL [134]. PROTAC‐based BTK degraders (e.g., NX‐2127, NX‐5948, HSK‐5948, GBG‐16673, ABBV‐101) selectively degrade BTK via the ubiquitin–proteasome system, offering a strategy against kinase‐inactive or gatekeeper mutants [83, 135, 136, 137, 138, 139, 140]. These agents are in phase 1 trials for R/R B‐cell malignancies (Table 2).

Taken together, inhibitors targeting the ATP‐binding pocket have reshaped the treatment of B‐cell malignancies; however, the emergence of multisite resistance mutations underscores the need for more adaptable therapeutic approaches. Early clinical data supporting dual‐mechanism inhibitors (e.g., LP‐168), BTK/LYN co‐targeting agents, and BTK degraders suggest promising strategies to address resistance after prior BTK‐directed therapies. By targeting both kinase‐dependent and kinase‐independent mechanisms, these next‐generation approaches may provide a path toward more durable disease control.

### 3.4 Safety Profiles and Class‐Specific Toxicities

While BTK inhibitors have fundamentally transformed the therapeutic landscape of B‐cell malignancies, their safety profiles remain a critical determinant of long‐term treatment sustainability. Adverse events associated with BTK inhibition can be broadly classified into on‐target effects, related to the physiological role of BTK in platelet activation and innate immune signaling, and off‐target toxicities arising from the inhibition of structurally related kinases, including TEC, ITK, epidermal growth factor receptor (EGFR), and members of the ERBB family [141, 142]. Notably, emerging pharmacokinetic–pharmacodynamic data suggest that toxicity is influenced not only by kinase selectivity but also by drug exposure and dosing intensity, underscoring the importance of individualized therapeutic strategies.

#### 3.4.1 Cardiovascular Toxicities

Cardiovascular complications represent the most clinically significant and well‐characterized toxicities associated with BTK inhibitors, particularly first‐generation agents. Atrial fibrillation (AF) and hypertension are the predominant manifestations. Ibrutinib, a first‐in‐class inhibitor with relatively broad kinase activity, has been consistently associated with AF rates of approximately 12%–16% in clinical trials and real‐world cohorts [142, 143]. Mechanistically, this risk has been attributed to off‐target inhibition of cardiac phosphoinositide 3‐kinase (PI3K)–Akt signaling and C‐terminal Src kinase (CSK), leading to impaired atrial electrical stability and increased arrhythmogenic susceptibility [144].

In contrast, second‐generation BTK inhibitors with improved selectivity profiles demonstrate significantly reduced cardiovascular toxicity. In the phase 3 ALPINE trial [6], zanubrutinib was associated with a markedly lower incidence of AF compared with ibrutinib (5.2% vs. 13.3%), and similar findings were observed with acalabrutinib in the ELEVATE‐RR study [101]. Noncovalent BTK inhibitors, such as pirtobrutinib, further minimize off‐target kinase inhibition and have demonstrated very low rates of AF (<3%), suggesting a potentially safer option for patients with preexisting cardiovascular comorbidities [111, 145].

Hypertension has emerged as another clinically relevant toxicity, with cumulative incidence exceeding 20%–25% in long‐term follow‐up studies [6, 101, 146]. Importantly, treatment‐emergent hypertension has been associated with an increased risk of major adverse cardiovascular events, highlighting the need for proactive monitoring and management.

#### 3.4.2 Hematological and Immunological Risks

On‐target inhibition of BTK in platelets disrupts glycoprotein VI‐mediated signaling and impairs primary hemostasis, resulting in an increased incidence of bleeding events. These are predominantly low‐grade manifestations, such as ecchymosis or petechiae, while major hemorrhage remains relatively uncommon (approximately 3%–6%) across different agents [6, 101, 145].

Hematologic toxicities, particularly neutropenia, are frequently observed and may vary across agents. Zanubrutinib, for example, has been associated with a higher incidence of grade ≥3 neutropenia (approximately 18%–22%), potentially reflecting its more complete and sustained BTK occupancy [6]. Beyond cytopenias, BTK inhibition exerts broader immunomodulatory effects by impairing macrophage and neutrophil function.

Consequently, patients receiving BTK inhibitors are at increased risk for opportunistic infections, including invasive fungal infections (e.g., Aspergillus species), Pneumocystis jirovecii pneumonia, and viral reactivation [6, 147]. Long‐term therapy may also lead to hypogammaglobulinemia, further compromising humoral immunity and predisposing patients to recurrent infections [148]. These findings underscore the importance of longitudinal immune monitoring and, in selected patients, prophylactic strategies.

#### 3.4.3 Gastrointestinal and Dermatological Effects

Gastrointestinal and dermatological toxicities are primarily driven by off‐target inhibition of EGFR signaling in epithelial tissues [149]. These effects are most commonly observed with ibrutinib and include diarrhea, rash, and mucosal irritation, reflecting disrupted epithelial homeostasis and turnover.

Second‐generation BTK inhibitors exhibit improved tolerability in this regard; however, they are associated with distinct class‐specific adverse effects. Acalabrutinib, for instance, is frequently associated with headache (incidence approximately 20%–40%), which is typically transient and may be related to vasodilatory effects on meningeal vessels [101]. Gastrointestinal symptoms such as diarrhea are often dose‐dependent and tend to attenuate over time, likely reflecting physiological adaptation or acquired tolerance. Although generally low grade (grade 1–2), these toxicities can significantly affect quality of life and treatment adherence, underscoring the need for early symptom management.

#### 3.4.4 Clinical Management and Emerging Therapeutic Implications

Effective management of BTK inhibitor‐associated toxicities requires a comprehensive and multidisciplinary approach. Baseline cardiovascular evaluation, including electrocardiography and assessment of preexisting risk factors, is recommended before treatment initiation. During therapy, a structured “hold–reduce–resume” strategy is commonly employed for grade 3 or higher nonhematologic adverse events. For patients who develop atrial fibrillation, management should include standard rate or rhythm control strategies in conjunction with individualized anticoagulation decisions, balancing the competing risks of thrombosis and bleeding [144]. Aggressive control of modifiable cardiovascular risk factors, particularly hypertension, is essential to mitigate long‐term complications. Switching between BTK inhibitors is an established strategy for managing treatment intolerance. Transitioning from first‐generation to more selective second‐generation or noncovalent inhibitors has been shown to improve tolerability while maintaining therapeutic efficacy [150]. However, abrupt discontinuation of BTK inhibition may precipitate disease flare or rapid progression in certain patients, emphasizing the importance of carefully planned transition strategies [151, 152].

Beyond toxicity management, the immunomodulatory properties of BTK inhibitors have expanded their therapeutic scope. By suppressing proinflammatory cytokine signaling and modulating myeloid cell activation, these agents are being investigated as adjunctive therapies to mitigate cytokine release syndrome in chimeric antigen receptor T‐cell therapy [153, 154]. Furthermore, their ability to modulate B‐cell and macrophage function has spurred interest in nonmalignant indications, including immune thrombocytopenia and other autoimmune disorders [153].

Collectively, the evolving safety profiles of BTK inhibitors reflect a dynamic interplay between kinase selectivity, pharmacologic exposure, and patient‐specific risk factors. As the field advances, optimizing toxicity management through precision‐based approaches will be essential to fully realize the long‐term benefits of continuous BTK inhibition therapy.

## 4 Applications of BTK Inhibitors in Hematologic Malignancies and Solid Tumors

The clinical development of BTK inhibitors has evolved from targeted suppression of malignant B‐cell survival signals toward a broader therapeutic strategy that integrates direct antitumor activity, immune modulation, and TME remodeling. In B‐cell malignancies, BTK inhibition disrupts BCR‐dependent proliferation, survival, adhesion, and tissue homing, thereby establishing the rationale for monotherapy across multiple disease settings. However, accumulating evidence indicates that the therapeutic effects of BTK inhibitors extend beyond malignant B cells. By modulating T‐cell function, immune checkpoint expression, myeloid‐derived suppressive signals, cytokine release, and stromal interactions, BTK inhibitors can enhance antitumor immunity and provide a mechanistic foundation for rational combination therapies. This section examines how BTK inhibitor monotherapy, immune modulation, TME remodeling, and combination strategies interact to improve therapeutic depth and durability in B‐cell malignancies, and further discusses the emerging relevance of BTK inhibition in solid tumors, where aberrant BTK isoforms (particularly p65BTK), microenvironmental crosstalk, and therapeutic resistance may open new avenues for BTK‐targeted intervention.

### 4.1 BTK Inhibitors Monotherapy in B‐Cell Malignancies

BTK is a central signaling node in the BCR pathway, and its inhibition has demonstrated robust and reproducible single‐agent activity across multiple mature B‐cell malignancies [83], particularly in chronic lymphocytic leukemia/small lymphocytic lymphoma (CLL/SLL), where it has redefined the treatment paradigm [155]. Covalent BTK inhibitors (ibrutinib, acalabrutinib, zanubrutinib) bind irreversibly to C481, sustaining BCR pathway blockade and suppressing downstream NF‐κB and PLCγ2 survival signals. This mechanism disrupts tumor–microenvironment crosstalk and induces nodal shrinkage with transient lymphocytosis from cellular redistribution [9, 156]. Clinically, these agents achieve high objective response rates (ORRs) and prolong progression‐free survival (PFS) in frontline and relapsed/refractory settings, replacing chemoimmunotherapy as the standard of care in selected disease contexts [157, 158].

Beyond CLL/SLL, BTK inhibitors are an established therapeutic option in other B‐cell malignancies, with the most mature data available in MCL and marginal zone lymphoma (MZL). Biologically, MCL is highly dependent on chronic BCR signaling and constitutive NF‐κB activation. Single‐agent BTK inhibition yields high response rates, durable disease control, and favorable tolerability, including in high‐risk patients with adverse features such as a Ki‐67 index ≥50% [159]. Clinical studies of acalabrutinib have reported PFS rates exceeding 80%, complete response (CR) rates of approximately 40%, and a median PFS exceeding 20 months [160]. MRD negativity has been achieved in approximately 28% of patients, an important prognostic indicator [161]. Additionally, the third‐generation noncovalent inhibitor pirtobrutinib has demonstrated clinically meaningful activity in patients previously exposed to covalent BTK inhibitors, with response rates of 50%–60%, including those harboring BTK C481 mutations [112, 162].

MZL is a heterogeneous malignancy arising from memory B cells and is strongly associated with antigen‐driven BCR activation in the setting of chronic infection or autoimmunity, supporting BTK as a rational therapeutic target. Zanubrutinib received FDA approval in 2021 for relapsed/refractory MZL [163]. In a multicenter study with a median follow‐up of 15.9 months, zanubrutinib achieved an ORR of 74.2%, a CR rate of 25.8%, and a 15‐month PFS rate of 82.5%, with a lower incidence of severe adverse events compared with ibrutinib [164].

In Waldenström macroglobulinemia (WM), the therapeutic relevance of BTK inhibition is closely linked to disease biology, as MYD88 and CXCR4 mutations drive dependence on BTK–NF‐κB signaling [165]. In patients with relapsed or refractory (R/R) WM, ibrutinib induces rapid and deep clinical responses, reducing bone marrow involvement from 60% to 25% and achieving an ORR of 90.5%. The greatest benefit is observed in patients harboring MYD88^L265P^/CXCR4^WT^ mutations, whereas responses are lowest in MYD88 wild‐type disease [165]. Another study further demonstrated that patients with wild‐type CXCR4 exhibit faster and higher major response rates than those with CXCR4 mutations, with a 4‐year PFS rate of up to 76%, indicating durable and effective disease control with BTK inhibition [166]. Nevertheless, the strong dependence of therapeutic efficacy on MYD88 and CXCR4 mutational status limits the applicability of ibrutinib to a subset of patients [167]. In contrast, the next‐generation BTK inhibitor zanubrutinib has demonstrated superior efficacy, a more favorable safety profile, and consistent clinical activity across molecular subtypes of WM [168]. Other BTK inhibitors under clinical investigation for WM include acalabrutinib and tirabrutinib [169].

In contrast, the activity of BTK inhibitor monotherapy in follicular lymphoma (FL) and R/R diffuse large B‐cell lymphoma (DLBCL) is limited. In relapsed FL, a phase II study of ibrutinib reported an ORR of approximately 37%, a CR rate of 12%, and a median PFS of 14 months, outcomes inferior to those observed in CLL or MZL [170]. In DLBCL, the PHOENIX trial comparing ibrutinib plus R‐CHOP with R‐CHOP alone in newly diagnosed non‐GCB patients failed to improve event‐free or overall survival and was associated with increased toxicity in older patients [171]. These findings underscore important safety considerations in clinical practice. The addition of BTK inhibitors to intensive chemoimmunotherapy regimens has been associated with a higher incidence of serious adverse events, including infections and gastrointestinal toxicities, particularly in patients aged 60 years or older. This age‐related increase in toxicity may reflect reduced physiological reserve and diminished tolerance to multiagent therapy, which can lead to dose modifications or treatment discontinuation. Such treatment interruptions may in turn attenuate the potential therapeutic benefit of combination strategies. Accordingly, in older patients, incorporating BTK inhibitors into combination regimens requires careful assessment of treatment‐related risks and close clinical monitoring to optimize the balance between efficacy and tolerability.

Despite the limited activity of BTK inhibitors in most DLBCL subtypes, an important exception is PCNSL, which predominantly exhibits an ABC phenotype. Ibrutinib monotherapy has produced ORRs of approximately 70% and CR rates near 50% in relapsed/refractory disease, with some patients achieving durable remissions [107, 172, 173]; indeed, ibrutinib demonstrates superior efficacy in relapsed or refractory CNS B‐cell lymphoma compared with peripheral B‐cell lymphoma [174]. Tirabrutinib has also shown clinical activity and was approved in Japan in 2020 for relapsed/refractory PCNSL, achieving an ORR of 63.6% and a median PFS of 2.9 months with a favorable safety profile [107]. Overall, BTK inhibitors show limited single‐agent activity in DLBCL, largely restricted to specific molecular subtypes, and BTK pathway inhibition is therefore more likely to serve as a component of combination strategies rather than as a standalone therapeutic backbone [155].

Despite these advances, the need for continuous BTK inhibitor therapy leads to cumulative drug exposure, raising concerns about long‐term safety and tolerability and underscoring the clinical relevance of treatment‐related adverse events. Adverse events associated with BTK inhibitors result from both on‐target and off‐target effects [175, 176]. While on‐target BTK inhibition underlies therapeutic efficacy, first‐generation agents such as ibrutinib exhibit broader off‐target activity against kinases including ITK, BMX, TEC, BLK, JAK3, and VEGFR2 due to structural homology [22, 177, 178]. These off‐target effects contribute to clinically relevant toxicities, including bleeding due to impaired platelet function [179, 180], atrial fibrillation potentially mediated through ERBB2/4 signaling [181, 182], and gastrointestinal disturbances associated with EGFR inhibition [183, 184]. In contrast, second‐generation BTK inhibitors such as acalabrutinib and zanubrutinib demonstrate improved selectivity, sparing ITK and EGFR while reducing TEC and BMX inhibition [100, 185], which translates into lower rates of cardiovascular, infectious, and gastrointestinal adverse events, although certain class‐specific toxicities such as headache remain more common [102, 186].

Taken together, BTK inhibitor monotherapy has established a new standard of care in several B‐cell malignancies; however, limitations in response depth, resistance, and long‐term safety underscore the need for next‐generation agents and rational combination strategies to achieve deeper and more durable disease control.

### 4.2 Mechanistic Basis of Combination Strategies With BTK Inhibitors

To provide a structured mechanistic overview, preclinical studies of BTK inhibitor–based combination strategies are categorized according to their dominant biological pathways and functional consequences (Table 3). Enhancement of T‐cell function, suppression of immune evasion, and remodeling of the TME represent the principal drivers of therapeutic synergy.

**TABLE 3:** Mechanism‐based summary of preclinical studies of BTK inhibitor combinations in B‐cell malignancies.

Mechanistic category	Combination strategy	Experimental model	Mechanisms	Functional consequence	Ref
Enhancement of T‐cell function	BTKi + CAR‐T	CLL, MCL, DLBCL (cell lines, primary samples, co‐culture systems, murine models, PDXs)	ITK inhibition‐driven Th1 polarization, activation of JAK‐STAT and PI3K/AKT/mTOR signaling, attenuation of exhaustion signaling including PD‐1 and TIM‐3, and transcriptional reprogramming toward a central memory phenotype with increased CD62L and reduced terminal differentiation	Enhanced CAR‐T expansion, persistence, trafficking, and cytotoxicity; improved tumor clearance and durable antitumor responses	[193, 197, 198]
	BTKi + ICI	Murine lymphoma models (A20)	Restoration of CD8^+^ T‐cell activation and cytokine production through reversal of exhaustion‐associated signaling	Improved T‐cell effector function and enhanced responsiveness to immune checkpoint blockade	[199, 200]
	BTKi + CD20×CD3bsAb	Primary CLL samples and PDXs	Improved T‐cell fitness, enhanced immune synapse formation, reversal of immunosuppression	Increased T cell‐mediated cytotoxicity independent of CD20 level; reduced tumor burden in vivo	[201]
Suppression of immune checkpoints and immune evasion	BTKi + CAR‐T	Co‐culture and murine models	Downregulation of inhibitory receptors including PD‐1, TIM‐3, CTLA‐4, and LAG‐3	Reduced CAR‐T exhaustion and maintained cytotoxic activity	[10, 188, 197]
	BTKi + ICI	CLL, DLBCL models	Inhibition of BCR/NF‐κB, STAT3, and NFATc1 signaling, reducing PD‐L1, IL‐10, and CD200 transcription	Attenuation of immune suppression and increased sensitivity to PD‐1/PD‐L1 blockade	[202, 203]
Remodeling of TME	BTKi + CAR‐T	CLL and lymphoma models	Reduction of myeloid‐derived suppressor cells, regulatory T cells, and M2‐polarized macrophages, together with decreased production of immunosuppressive cytokines	Improved immune cell infiltration and enhanced CAR‐T efficacy	[198]
	BTKi targeting MDSCs	Murine models	Inhibition of BTK‐dependent nitric oxide and IDO production	Reversal of MDSC‐mediated T‐cell suppression	[204, 205]
	BTKi targeting TAMs	CLL and lymphoma models	Inhibition of M2 polarization and suppression of NLRP3 inflammasome activation and IL‐1β production	Reduced tumor‐promoting inflammation and impaired tumor–macrophage interactions	[206, 207, 208]
	BTKi + metabolic modulation	TME models	Regulation of cytokine‐driven metabolic pathways and interaction with PD‐1 signaling	Restoration of T‐cell metabolic fitness and reversal of immune exhaustion	[209, 210]
	BTKi targeting Tregs/Th17	In vitro and in vivo	Modulation of T‐cell differentiation through ITK‐dependent and ITK‐independent mechanisms	Reduced regulatory T‐cell activity and shift toward a pro‐inflammatory immune state	[211]
Inhibition of tumor cell homing and retention	BTKi + CD20 mAb/CAR‐T/BCL2 inhibitors	CLL, MCL, WM models; in vivo and murine models	Inhibition of CXCR4/CXCL12 and CXCR5 signaling, suppression of integrin‐mediated adhesion, and reduced production of chemokines such as CCL3 and CCL4, together with concurrent inhibition of BCR‐related pathways including BTK, SYK, and PI3Kδ, leading to disruption of chemokine‐driven retention signals	Mobilization of tumor cells from protective niches into circulation and impaired migration, enhancing therapeutic accessibility and immune infiltration	[212, 213]
Mitigation of CRS and immune toxicity	BTKi + CAR‐T	Co‐culture and murine models	Suppression of myeloid‐derived cytokine production, including IL‐6, TNF‐α, and IL‐1β, through modulation of TLR and NLRP3 signaling pathways, together with reduction of inflammatory myeloid populations and regulation of cytokine kinetics and immune activation balance	Reduced cytokine release and attenuated CRS severity without compromising CAR‐T expansion or cytotoxicity	[153]

Abbreviations: BTKi, Bruton's tyrosine kinase inhibitor; CAR‐T, chimeric antigen receptor T cell; ICI, immune checkpoint inhibitor; CLL, chronic lymphocytic leukemia; DLBCL, diffuse large B‐cell lymphoma; MCL, mantle cell lymphoma; PDX, patient‐derived xenograft; CRS, cytokine release syndrome; MDSC, myeloid‐derived suppressor cell; TAM, tumor‐associated macrophage; Treg, regulatory T cell.

#### 4.2.1 BTK Inhibitors Enhance T Cell Function

CAR‐T therapy requires the collection of T cells from patients; however, these T cells often exhibit dysfunction due to damage inflicted by the tumor and prior treatments, leading to insufficient CAR‐T cell quantity and reduced quality. T‐cell dysfunction is particularly severe in patients with CLL, characterized by an increased proportion of terminally differentiated phenotypes and a reduction in naïve T cells, which severely limits CAR‐T cell expansion and functional efficacy [187]. The BTK inhibitor ibrutinib has demonstrated significant immunomodulatory effects in improving T‐cell immune status. Preclinical and translational studies have shown that prolonged ibrutinib treatment restores T‐cell responsiveness to T‐cell antigen receptor (TCR) stimulation and enhances CAR‐T cell expansion, persistence, in vivo trafficking, and antitumor activity [188]. Similarly, acalabrutinib and orelabrutinib have been reported to increase CAR‐T cell proliferative capacity and cytotoxicity, thereby contributing to reduced tumor burden [189, 190, 191].

In CLL, immune polarization is frequently skewed toward a Th2‐dominant phenotype associated with disease progression [192]. ITK, a member of the TEC kinase family, plays a central role in proximal TCR signaling and in the differentiation and maturation of T lymphocytes [2]. In addition to ITK, resting lymphocyte kinase (RLK) is also expressed on Th1 and CD8^+^ T lymphocytes, contributing to cell activation [193]. In Th1‐polarized and CD8^+^ T cells, ITK supports but does not critically determine RLK function. However, ITK maintains the sole dominant role in the epigenetic evolution of Th2 cells, making it a critical vulnerability for Th2 cells [194, 195]. ITK exhibits significant sequence and functional homology with BTK [196]. Due to this homology, ibrutinib can irreversibly inhibit ITK through off‐target activity, suppressing Th2 polarization and promoting a Th1 phenotype characterized by increased IFN‐γ and IL‐2 production and enhanced cytotoxicity [193] (Figure 4A). Next‐generation BTK inhibitors exhibit minimal ITK inhibition yet still improve CAR‐T cell efficacy, suggesting the involvement of additional ITK‐independent mechanisms [81, 189, 197].

**FIGURE 4:**
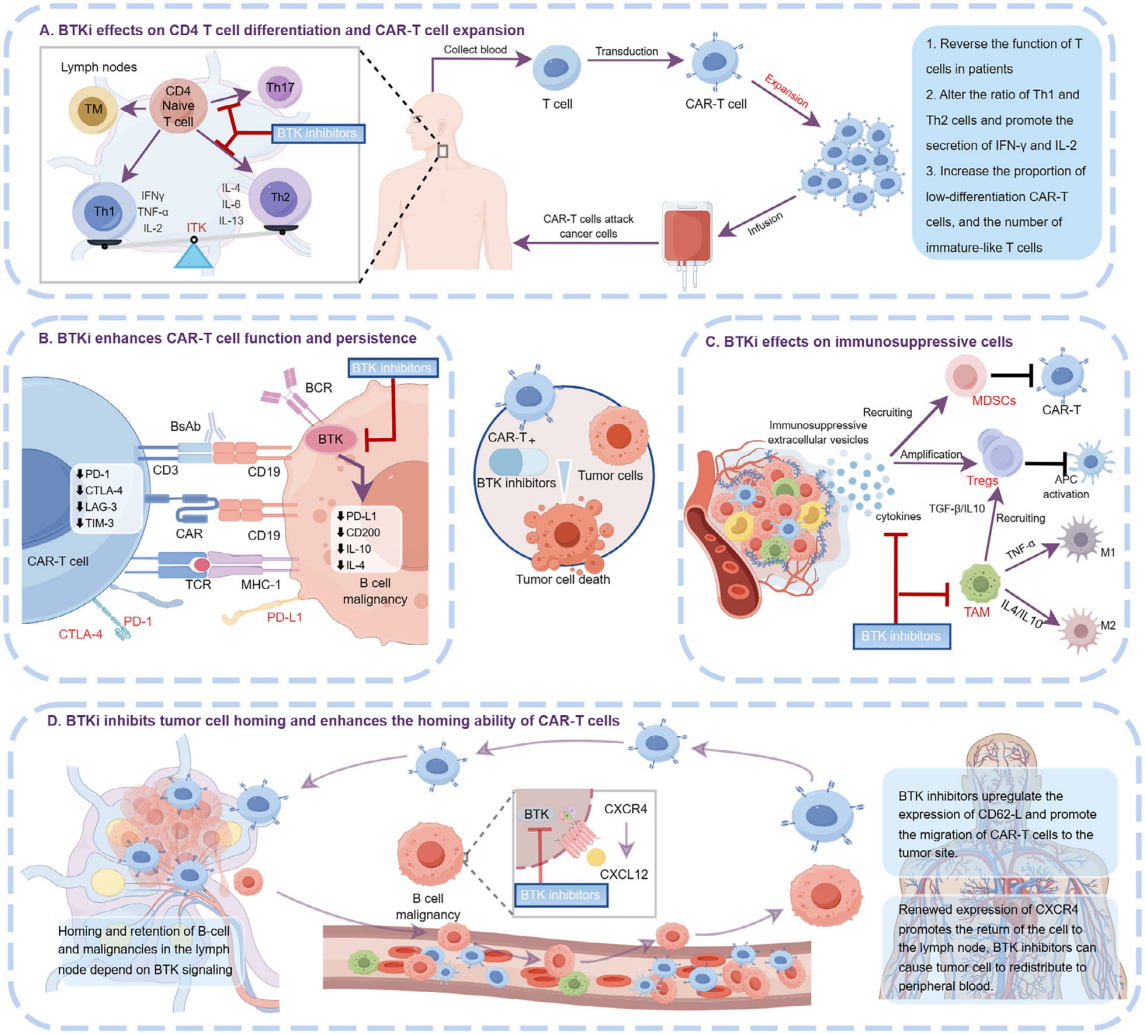
Mechanisms underlying combination strategies of BTK inhibitors with CAR‐T cell therapy. (A) Effects on CD4+ T‐cell differentiation: BTK inhibitors irreversibly inhibit ITK via off‐target effects, selectively suppressing Th2 polarization and shifting the Th1/Th2 balance; they also alter T‐cell subset composition by increasing memory T cells and downregulating Th17 cells. (B) Enhancement of CAR‐T cell function and persistence: BTK inhibitors reduce CAR‐T cell exhaustion and enhance antitumor activity by decreasing expression of PD‐1, CTLA‐4, and other immune checkpoints, blocking PD‐1/PD‐L1 interaction, and attenuating TME‐mediated negative regulation. (C) Suppression of immunosuppressive cells: BTK inhibitors directly or indirectly suppress MDSCs, Tregs, and TAMs while reducing secretion of inhibitory cytokines such as IL‐10, thereby diminishing TME immunosuppression. (D) Inhibition of tumor cell homing: BTK inhibitors downregulate CXCR4 or CXCR5 expression and inhibit downstream signaling, preventing tumor cell homing to lymph nodes and promoting mobilization into peripheral blood for recognition and elimination by CAR‐T cells.

BTK inhibition also reshapes T‐cell composition and transcriptional programs. Ibrutinib induces significant alterations in T‐cell subsets, including increased proportions of CD4^+^ and CD8^+^ T cells, expansion of effector memory T cells, and reduced regulatory T‐cell frequency [197]. BTK expression has been detected in T cells, particularly within effector and memory subsets, suggesting a potential role in T‐cell activation and indicating that BTK inhibitors may exert direct immunomodulatory effects independent of tumor targeting [214]. At the transcriptional level, long‐term ibrutinib treatment significantly alters the gene expression profile of CD19 CAR‐T cells [189], downregulating granzyme A (GZMA) while upregulating SELL (CD62L), a marker associated with central memory T cells. Concurrently, MSC, which promotes Th1 differentiation, is upregulated, whereas Th2‐related genes such as NRIP1, LZTFL1, and RARRES3 are suppressed. These changes suggest that ibrutinib promotes a memory‐like phenotype while limiting terminal differentiation. Consistent with this, transcriptomic analysis of CAR‐T cells co‐cultured with vecabrutinib revealed marked upregulation of genes involved in the PI3K/AKT/mTOR and Th1 pathways [215]. Functionally, BTK inhibitors impair terminal T‐cell differentiation while promoting memory T‐cell development, thereby enhancing in vivo persistence and sustained antitumor efficacy [191].

In the setting of CAR‐T cell exhaustion, BTK inhibitors attenuate chronic signaling and persistent antigenic stimulation [198], partly through modulation of pathways such as JAK‐STAT [190]. Future studies should aim to elucidate the mechanisms by which BTK inhibitors modulate CAR‐T cell function and to develop more effective treatment strategies for patients with B‐cell malignancies.

Beyond CAR‐T modulation, BTK inhibitors enhance the efficacy of immune checkpoint inhibitors. Preclinical studies have shown that although PD‐1/PD‐L1 blockade alone may delay CLL progression, its antitumor effect is significantly amplified when combined with ibrutinib [199]. This combination increases the proportion of effector CD8^+^ T cells, upregulates activation markers, and enhances degranulation and IFN‐γ production, collectively restoring T‐cell effector function [199]. In A20 lymphoma mouse models, BTK inhibition converts weak antitumor T‐cell responses into robust immune activity, improving survival even in tumors insensitive to BTK inhibitor monotherapy [200].

Together, these findings underscore the dual immunopotentiating roles of BTK inhibitors in correcting T‐cell dysfunction and augmenting both CAR‐T and immune checkpoint inhibitor responses. Such immunomodulatory properties provide a strong rationale for combining BTK inhibitors with immunotherapies to improve outcomes in B‐cell malignancies.

#### 4.2.2 BTK Inhibition Downregulates Immune Checkpoints to Enhance Antitumor Immunity

The upregulation of immune checkpoints, including programmed cell death protein 1 (PD‐1), programmed cell death ligand 1 (PD‐L1), CTLA‐4, and T‐cell immunoglobulin and mucin domain‐containing protein 3 (TIM‐3), on tumor cells and tumor‐infiltrating lymphocytes (TILs) constitutes a key mechanism of immune evasion in B‐cell malignancies [216]. PD‐L1 is highly expressed on both tumor cells and tumor‐associated immune cells such as TAMs [217] and is closely associated with poor prognosis in lymphomas including DLBCL and HL [218]. Aberrant PD‐L1 expression contributes to CAR‐T cell exhaustion and impaired cytotoxicity, promoting relapse after CAR‐T therapy and resistance to CD20‐targeted treatments [219, 220]. Additionally, PD‐L1 signaling promotes regulatory T‐cell (Treg) differentiation and FOXP3 expression, reinforcing Treg‐mediated immunosuppression [221]. Together with TAMs, Tregs suppress effector T‐cell proliferation and cytotoxicity, establishing an immunosuppressive TME [222].

CD200 is another immunoregulatory molecule frequently overexpressed in hematologic malignancies [223]. Engagement of CD200 with its receptor CD200R suppresses myeloid cell activation [224], shifts cytokine profiles from Th1 (IFNs, IL‐2) toward Th2 (IL‐10, IL‐4) [225], and inhibits T‐cell and NK‐cell cytotoxic responses [226, 227]. CD200 can also be released in soluble form following metalloproteinase cleavage, binding CD200R on antigen‐presenting cells to promote expansion of MDSCs and Tregs while suppressing IL‐2 and IFN‐γ secretion [228].

Mechanistically, BTK inhibition suppresses key oncogenic and immunoregulatory pathways, including BCR/NF‐κB, STAT3 [188], and NFATc1, resulting in reduced transcription of PD‐L1, IL‐10, and CD200 [202, 203]. Both BCR and CD40 signaling pathways induce IL‐10 production, which in turn activates the JAK/STAT pathway through autocrine signaling. PD‐L1 expression is therefore regulated either directly by CD40‐mediated NF‐κB activation or indirectly through IL‐10‐induced BCR sensitization and subsequent BTK activation.

In preclinical studies, BTK inhibitors significantly reduced the expression of PD‐1, CTLA‐4, TIM‐3, CD200, and BTLA on tumor cells and T cells, along with decreased secretion of immunosuppressive cytokines such as IL‐10 and IL‐4 [204, 206, 229, 230] (Figure 4B). In the context of CAR‐T cell therapy, BTK inhibitors help preserve T‐cell cytotoxicity by preventing immune exhaustion. Co‐culture studies with malignant B cells demonstrated that ibrutinib reduced PD‐1, TIM‐3, CTLA‐4, and LAG‐3 expression on CAR‐T cells while enhancing their persistence and tumor control [10]. Similar effects have been reported for acalabrutinib and zanubrutinib, which decreased CD200, PD‐1, CTLA‐4, CD160, and BTLA expression and suppressed IL‐10 production in T cells [197, 231]. These immunosuppressive markers correlate inversely with CAR‐T cell expansion ex vivo, and BTK inhibition correspondingly enhances CAR‐T19 cytotoxicity while reducing PD‐1, TIM‐3, and CTLA‐4 mRNA levels [190].

Unlike ibrutinib, the highly selective BTK inhibitor pirtobrutinib exhibits minimal ITK inhibition, suggesting that its synergy with PD‐1/PD‐L1 blockade may arise primarily from on‐target BTK inhibition in malignant B cells and myeloid populations rather than ITK‐mediated T‐cell polarization [88]. This distinction warrants further evaluation through detailed immune phenotyping.

ICIs, particularly monoclonal antibodies targeting PD‐1 or PD‐L1, block the immunosuppressive PD‐1/PD‐L1 axis and reactivate cytotoxic T‐cell‐mediated antitumor activity [199, 232], representing a cornerstone of contemporary cancer immunotherapy. Similarly, combining BTK inhibitors with ICIs offers a synergistic strategy to overcome the immunosuppressive TME. In DLBCL, BTK inhibition reduces PD‐L1 expression by disrupting IL‐10‐mediated feedback and BCR/CD40 crosstalk, thereby enhancing sensitivity to PD‐1/PD‐L1 blockade [202, 203]. Combination therapy may also restore T‐cell function in ICI‐resistant settings; for example, ibrutinib plus nivolumab has demonstrated clinical activity in relapsed or refractory classical Hodgkin lymphoma after failure of anti‐PD‐1 monotherapy [163].

BTK inhibition enhances T‐cell recognition and cytotoxicity against tumor cells. When combined with anti‐CD20 monoclonal antibodies, this immunomodulatory effect further augments ADCC and ADCP. However, off‐target ITK inhibition by ibrutinib may impair rituximab‐mediated NK‐cell ADCC [233, 234, 235]. In contrast, the highly selective BTK inhibitor orelabrutinib has demonstrated robust clinical activity in R/R CLL and MCL without significantly suppressing NK cell‐mediated ADCC [236]. Its antitumor efficacy is further enhanced when combined with anti‐CD20 antibodies compared with monotherapy, suggesting that next‐generation selective BTK inhibitors preserve ADCC function and may serve as a more effective backbone for combination strategies with CD20‐directed therapies.

In summary, BTK inhibitors downregulate PD‐1/PD‐L1, CTLA‐4, and other immunosuppressive molecules, improve responsiveness to immune checkpoint blockade, and enhance the efficacy of cellular immunotherapy and targeted therapies. Collectively, these mechanisms provide a strong biological rationale for BTK inhibitor‐based combination approaches to improve outcomes in B‐cell malignancies.

#### 4.2.3 BTK Inhibitors Reshape the TME by Targeting Immunosuppressive Cells

The TME in B‐cell malignancies comprises a complex network of immune and stromal cells that supports tumor progression and suppresses effective antitumor immunity. These cells promote B‐cell lymphoma development through the secretion of cytokines and chemokines [237]. Among them, immunosuppressive components such as myeloid‐derived suppressor cells (MDSCs), Tregs, and TAMs restrict T‐cell infiltration and function, thereby limiting the efficacy of immunotherapy [238, 239]. In this context, the immunosuppressive TME can impair CAR‐T cell expansion, infiltration, and persistence, ultimately compromising therapeutic outcomes [240]. Empirical evidence indicates that administering BTK inhibitors before CAR‐T cell infusion can effectively reduce tumor burden and improve both immune function and the microenvironmental milieu [189]. This intervention may counteract CAR‐T cell dysfunction, delay exhaustion, or promote expansion. Notably, the combination of ibrutinib with CD19 CAR‐T cells demonstrated significant efficacy in a subcutaneous tumor model but proved ineffective in a tail vein model [198, 241]. These observations suggest that the therapeutic benefit of BTK inhibitors may be mediated, at least in part, by TME remodeling, thereby enhancing the effectiveness of CAR‐T cell therapy.

MDSCs are classified into two major subsets: polymorphonuclear MDSCs (PMN‐MDSCs) and monocytic MDSCs (M‐MDSCs). Both subsets suppress T‐cell function and impair CAR‐T cell infiltration and persistence by generating reactive oxygen species (ROS), depleting essential nutrients such as arginine, and secreting immunosuppressive factors including arginase‐1 (ARG1) [237, 238]. Notably, an increased frequency of M‐MDSCs has been significantly associated with treatment failure of axi‐cel therapy [242]. In solid tumors, MDSC accumulation is considered a major barrier to effective CAR‐T cell infiltration [243]. Within the TME, MDSCs activate Tregs by secreting interleukin‐10 (IL‐10), indoleamine 2,3‐dioxygenase (IDO), and arginase‐1, while concurrently suppressing T‐cell and natural killer (NK) cell function [244].

BTK inhibition interferes with these suppressive mechanisms. Ibrutinib suppresses BTK phosphorylation in MDSCs, reducing nitric oxide production and cellular migration while decreasing expression of immunosuppressive mediators such as IDO [205] (Figure 4C). Second‐generation BTK inhibitors, including acalabrutinib and zanubrutinib, have similarly been reported to alleviate MDSC‐mediated suppression of CD8^+^ T cells, thereby improving T‐cell function without markedly altering immune cell composition [204, 205]. Additionally, combination strategies have been proposed. For instance, TR2‐41BB bispecific CAR‐T cells directly induce MDSC apoptosis and effectively restore CAR‐T antitumor activity [245] (Figure 4C).

Tregs inhibit T‐cell activation by obstructing CTLA‐4–mediated functional activation of antigen‐presenting cells (APCs) [246]. ITK has been implicated in the regulation of Tregs and Th17 cell differentiation, although the specific role of Th17 cells within the TME remains unclear [219]. In vitro studies have indicated that ibrutinib exerts a dose‐dependent effect on Tregs: low concentrations promote Treg expansion, whereas higher doses inhibit their proliferation [211]. Clinical data further suggest that ibrutinib treatment in patients with B‐cell malignancies is associated with a reduction in circulating Treg levels. In contrast, second‐generation BTK inhibitors such as acalabrutinib appear to lack this suppressive effect on Tregs [197, 247]. Additionally, ibrutinib inhibits Th17 differentiation both in vitro and in vivo through ITK‐dependent and ITK‐independent mechanisms [207]. Given that both Tregs and Th17 cells contribute to the immunosuppressive TME, these findings suggest that BTK inhibition may help restore a more immunostimulatory milieu (Figure 4C).

TAMs represent another critical target within the TME. Macrophage polarization strongly influences tumor immunity: M1‐type TAMs exhibit proinflammatory and antitumor properties, whereas M2‐type TAMs demonstrate immunosuppressive functions and inhibit T‐cell activity [248]. Patients with bulky disease (defined as tumor diameter [*D*_max_] > 4.0 cm) show significantly increased M2 macrophage infiltration compared with those without bulky masses [249]. TAMs are often skewed toward an M2 phenotype and secrete immunosuppressive cytokines such as IL‐10 and TGF‐β, which promote Treg recruitment and impair effector T‐cell function [250]. TAMs also express immune checkpoint molecules including PD‐L1 and CD200, contributing to resistance to CAR‐T therapy and immune checkpoint blockade [237].

BTK inhibitors modulate TAM function by inhibiting M2 polarization, enhancing phagocytic activity, and suppressing the production of immunosuppressive cytokines such as IL‐10 [190, 198]. BTK serves as a direct regulator of NOD‐like receptor protein 3 (NLRP3) inflammasome activation [206, 208]. Ibrutinib blocks cytokine‐mediated TAM migration to the TME, reduces NLRP3 activation and IL‐1β production, and thereby suppresses the tumor‐promoting activity of TAMs. In CLL, ibrutinib significantly reduces TAM‐derived CXCL13 expression in the bone marrow, impairing CLL cell homing and limiting their migration [207]. Ibrutinib also disrupts the TME by diminishing interactions between TAM pseudopods and CLL cells [207]. In summary, BTK inhibitors modulate TAM‐mediated immunosuppressive pathways by reducing chemokine and cytokine production, thereby impairing lymphoma cell adhesion, invasion, and migration, ultimately suppressing tumor progression and reducing therapy‐related resistance.

Metabolic competition within the TME, particularly for glucose, impairs aerobic glycolysis in tumor‐infiltrating lymphocytes (TILs) and tumoricidal macrophages [251, 252]. PD‐1 signaling exacerbates these functional impairments by suppressing glycolysis, phagocytosis, and BTK signaling. However, blockade of the PD‐1/PD‐L1 axis can reverse such immune‐metabolic dysfunction [209, 210]. Although BTK inhibition may further impair monocyte immune metabolism, its combination with ICIs provides a synergistic strategy to restore immune‐metabolic function within the TME and reverse immune exhaustion.

Overall, BTK inhibitors reshape the TME by targeting multiple immunosuppressive cell populations, including MDSCs, Tregs, and TAMs. Through modulation of cytokine signaling, immune checkpoints, and metabolic pathways, BTK inhibition alleviates immunosuppression and enhances the efficacy of immunotherapies in B‐cell malignancies.

#### 4.2.4 BTK Inhibitors Remodel the TME by Inhibition of Tumor Cell Homing and Aggregation

Aberrant activation of the BCR signaling pathway is a key driver in various B‐cell malignancies, promoting uncontrolled proliferation, impairing apoptosis and differentiation, and leading to the pathological accumulation of malignant B cells in the bone marrow, secondary lymphoid organs, and peripheral blood [48]. A hallmark of diseases such as CLL, MCL, and WM is the homing and retention of tumor cells within protective microenvironmental niches, including the bone marrow and lymph nodes [253, 254, 255]. Notably, approximately 30% of WM patients harbor the CXCR4 S338X mutation, which enhances CXCL12‐mediated activation of AKT and ERK signaling pathways, thereby facilitating tumor cell retention in the TME [256].

Chemokine‐regulated, integrin‐mediated trafficking plays a critical role in B‐cell homing and retention in CLL and MCL. BTK, a key component of BCR signaling, also mediates chemokine‐induced B‐cell trafficking through the CXCR4–CXCL12 axis. BTK inhibitors, such as ibrutinib and zanubrutinib, downregulate the expression of chemokine receptors including CXCR4 and CXCR5, thereby disrupting chemokine‐induced signaling cascades [9, 204, 212, 257] (Figure 4D). This results in impaired tumor cell homing to lymphoid tissues and promotes their redistribution into the peripheral blood, a phenomenon frequently observed in patients with CLL and also documented in MCL and other B‐cell lymphomas [60, 258, 259, 260]. This lymphocyte redistribution is considered a hallmark pharmacodynamic response to BTK inhibition, improving exposure to antibody‐based therapies and BCL2 inhibitors.

Disruption of tumor cell retention within lymphoid niches may also alleviate immunosuppressive pressure in the TME and enhance immune‐mediated tumor clearance. CAR‐T therapies have shown high efficacy in clearing bone marrow disease, but their effectiveness against bulky lymph node disease remains limited, likely due to impaired CAR‐T cell infiltration and function within the immunosuppressive lymph node milieu [9]. Supporting this, a study involving ibrutinib in B‐cell lymphoma demonstrated a reduction in lymph node volume alongside a transient increase in circulating malignant B cells, reinforcing BTK inhibition as a means to alter the spatial distribution of tumor cells [259]. Furthermore, combined inhibition of BTK, SYK, and PI3Kδ, key regulators of B‐cell trafficking, can synergistically impair tumor cell migration, pointing to a broader therapeutic strategy targeting tumor–TME interactions [55, 261].

Emerging clinical and translational evidence also supports the use of BTK inhibitors in conjunction with CAR‐T cell therapies. For instance, pirtobrutinib, a noncovalent, reversible BTK inhibitor, is currently under investigation as a bridging and maintenance agent in combination with the bispecific anti‐CD19/CD20 CAR‐T product LV20.19. Early data from a dedicated Phase 1 study (NCT05990465) suggest that pirtobrutinib (administered at 200 mg daily) is both feasible and safe in this setting, showing no adverse effects on T‐cell immunophenotype and a trend toward enhanced CAR‐T functionality. Favorable clinical responses and manageable toxicity were reported in early experiences, justifying further investigation [88, 262, 263].

Mechanistically, pirtobrutinib‐mediated BTK blockade suppresses BCR signaling and reduces expression of the chemokines CCL3 and CCL4, resulting in transient lymphocytosis consistent with tumor–microenvironment disengagement [91]. Moreover, during the peri‐infusion period, continued BTK inhibition is hypothesized to reduce myeloid‐derived cytokines such as IL‐6 and IL‐1, potentially mitigating cytokine release syndrome (CRS) and immune effector cell‐associated neurotoxicity syndrome (ICANS) without impairing CAR‐T cytotoxicity. These effects are consistent with the role of BTK in TLR and Fc receptor signaling in myeloid cells and are supported by preclinical and early clinical studies combining covalent BTK inhibitors with CAR‐T therapy [91].

Beyond CAR‐T‐based strategies, BTK inhibition may also enhance the efficacy of ICIs. Preclinical studies have demonstrated that inhibition of CXCR4 signaling can restore T‐cell responsiveness to PD‐1 and CTLA‐4 blockade [264]. In pancreatic cancer models, CXCR4 inhibitors induced rapid tumor cell accumulation and exhibited synergistic activity with PD‐L1 blockade, leading to significant reductions in tumor burden [265, 266]. Given that BTK operates downstream of the CXCR4–CXCL12 signaling axis, these findings provide a mechanistic rationale for combining BTK inhibitors with PD‐1/PD‐L1 inhibitors in immunotherapy regimens.

Importantly, the immunomodulatory effects of BTK inhibition vary among agents. For example, an in vitro study of CLL found that acalabrutinib monotherapy was more effective in reducing tumor burden than when combined with anti‐PD‐1 antibodies [267]. Histopathological analysis revealed increased lymph node involvement and a higher mitotic index in combination‐treated mice, suggesting potential tumor‐promoting effects associated with altered NF‐κB and PKC signaling [267]. These findings highlight the need to carefully consider the immunomodulatory profiles of specific BTK inhibitors when designing combination strategies.

Beyond cell therapy, pirtobrutinib is being explored in novel frontline regimens. An ongoing Phase II trial (NCT06333262) is evaluating a fixed‐duration doublet therapy of pirtobrutinib and obinutuzumab in previously untreated CLL. Additionally, a time‐limited triplet regimen of pirtobrutinib, venetoclax, and obinutuzumab has demonstrated promising efficacy, achieving undetectable minimal residual disease at 10^−^^6^ (uMRD6) rates of 80%–85% at 12 months. The tolerability profile remains consistent with individual agent expectations, with longer follow‐up planned to assess durability of response.

Beyond cellular immunotherapy, these microenvironmental changes also directly influence antibody‐mediated effector functions. As anti‐CD20 monoclonal antibodies primarily eliminate tumor cells through antibody‐dependent cellular cytotoxicity (ADCC) and myeloid phagocytosis, the immunomodulatory effects of BTK inhibition create a more favorable environment for effector cells, including T cells and NK cells [236]. This may augment both direct cytotoxicity and T cell‐mediated antitumor responses, reduce immune evasion, and provide a mechanistic rationale for the clinical synergy between BTK inhibitors and CD20‐targeted antibodies.

#### 4.2.5 BTK Inhibitors Mitigate CAR‐T Associated Cytokine Release Syndrome

CAR‐T cell therapy elicits potent antitumor responses by expanding and activating engineered T cells targeting CD19^+^ malignant B cells, while concurrently stimulating immune and endothelial cells [268]. However, this activation is frequently accompanied by CRS, a potentially life‐threatening complication characterized by the rapid and excessive release of proinflammatory cytokines such as IL‐6, IL‐8, TNF‐α, and IFN‐γ [268, 269]. Severe CRS can lead to systemic inflammation, respiratory failure, cardiovascular collapse, and multi‐organ dysfunction [270]. The risk of CRS is influenced by multiple factors, including disease characteristics, CAR structure, tumor burden, and the dose of CAR‐T cells administered [271].

Emerging evidence suggests that BTK inhibitors (BTKis) may mitigate CRS while preserving CAR‐T antitumor activity. Clinical data support this premise: patients receiving CAR‐T therapy after ibrutinib failure exhibited lower CRS rates and better therapeutic responses [26]. Clinical trials have confirmed that combining ibrutinib with CAR‐T therapy reduces the incidence of CRS in patients with CLL, and the overall efficacy is superior to that of CAR‐T monotherapy [272], suggesting a potential advantage of combination therapy.

Mechanistically, BTK inhibition appears to modulate cytokine production within the TME. In vitro, ibrutinib combined with CD19 CAR‐T cells significantly inhibits the release of IL‐2, IL‐6, TNF‐α, and IFN‐γ while enhancing T cell‐mediated cytotoxicity [272]. Studies using isolated TME models have further shown that BTK inhibitors can downregulate IL‐6 and TNF‐α levels and reduce the severity of CRS [190, 198, 273], an effect potentially attributable to their ability to suppress MDSC generation and TNF‐α secretion [205]. Animal model studies have also verified that combining ibrutinib with CD19 CAR‐T therapy reduces cytokine production without impairing T‐cell proliferation [153], although the specific mechanism remains unclear. Similarly, the noncovalent BTK inhibitor vecabrutinib has demonstrated enhanced antigen specificity of CAR‐T cells and a reduction in CRS‐associated cytokines, such as IL‐6, IL‐10, and MIP‐1β, findings that have been confirmed in phase 1 clinical trials [215].

Nevertheless, the relationship between BTK inhibition and CRS remains complex. Notably, some studies have reported that improved CAR‐T expansion and function may paradoxically increase cytokine levels. Clinical observations showed that patients with CRS had more robust peripheral CAR‐T cell expansion than those without CRS [272]. Mechanistic studies suggest that ibrutinib may simultaneously enhance CAR‐T cell function and cytokine release capacity [191]. For instance, in patients with R/R NHL who received ibrutinib as salvage therapy following the failure of initial anti‐CD19 CAR‐T therapy, increased CAR‐T cell activity was observed during subsequent CD19 CAR‐T therapy [274]. In a study of patients with R/R CLL, patients who received ibrutinib immediately before or during infusion showed significantly greater increases in IL‐6 and IL‐10 levels compared with those who did not receive ibrutinib [275], accompanied by increased hematologic toxicity and higher CRS grades. These conflicting findings highlight the dual role of BTKis in boosting CAR‐T function and modulating cytokine kinetics. The influence of timing, dosage, and disease context on this balance remains unclear and warrants further mechanistic and clinical investigation.

Overall, BTK inhibitors represent a promising adjunct to CAR‐T therapy through their capacity to modulate inflammatory cytokine signaling and reshape the immune microenvironment. Their ability to mitigate CRS while maintaining or enhancing CAR‐T efficacy supports their incorporation into rational combinatorial strategies for B‐cell malignancies. However, optimizing their clinical use requires a nuanced understanding of dose–response dynamics and context‐specific effects.

### 4.3 Clinical Advances in Combination Therapy With BTK Inhibitors in B‐Cell Malignancies

#### 4.3.1 BTK Inhibitors and CAR‐T Cell Therapy

The combination of CAR‐T therapy with BTK inhibitors is advancing from preclinical studies to clinical application. Multiple trials are underway, and early results are encouraging. In a study evaluating the safety and feasibility of co‐administering ibrutinib with CD19 CAR‐T cell therapy, ibrutinib was initiated at least two weeks before leukapheresis and continued for ≥3 months after infusion. Compared with CAR‐T monotherapy, the combination group demonstrated improved clinical outcomes, with an ORR of 83% and a bone marrow MRD‐negative rate of 61%. CRS severity was attenuated, and treatment was generally well tolerated [272]. Similarly, in a phase II trial involving high‐risk CLL patients with suboptimal responses to prolonged ibrutinib monotherapy, addition of CD19 CAR‐T therapy resulted in durable and deep remissions. The CR rate at 3 months was 44%, and by 12 months, 72% of evaluable patients had no detectable MRD. The estimated OS and PFS at 48 months were 84% and 70%, respectively. Importantly, this strategy demonstrated efficacy in genetically high‐risk populations, including TP53‐mutated CLL, suggesting potential to overcome adverse prognostic features. Notably, 63.2% of CRS events were grade 1, significantly reducing the incidence of grade 3–4 CRS, while ICANS incidence remained comparable to historical data [276].

A phase II study in MCL (*N* = 20) showed that continuous ibrutinib from pre‐leukapheresis through ≥6 months postinfusion achieved a 4‐month CR rate of 80% and 1‐year PFS and OS of 75% and 100%, respectively. CRS was predominantly low grade, with only 15% of patients experiencing grade 3 events. Efficacy was unaffected by prior BTK inhibitor exposure or TP53 mutation status, highlighting the robustness of this strategy in high‐risk populations [5]. These findings are supported by multicenter studies in BTKi‐exposed or refractory MCL, in which patients received optional BTKi‐based bridging but no concurrent BTK inhibition during CAR‐T therapy. In this setting, ORRs exceeded 90% with CR rates up to 67% and durable survival (1‐year OS approximately 83%) [277].

In a retrospective study of 21 patients with R/R DLBCL, a zanubrutinib‐based bridging regimen combined with CD19 CAR‐T therapy showed promising outcomes. Before cell infusion, 61.9% of patients experienced significant tumor burden reduction, with ≥50% shrinkage in the sum of the product of diameters (SPD) of target lesions. The median PFS was 12.8 months; 1‐year PFS and OS rates were 52.4% and 80.1%, respectively. CRS occurred in 85.7% (all grade 1–2); ICANS was observed in 9.5% (all grade 1), supporting the feasibility and efficacy of zanubrutinib bridging without compromising CAR‐T safety [231]. Additional real‐world and retrospective studies have further confirmed that zanubrutinib combined with CAR‐T therapy yields ORRs of approximately 80%–88% and CR rates up to 70%, with durable remissions and manageable toxicity profiles, even in patients with high‐risk features such as TP53 mutations or double‐hit lymphoma [278].

Emerging clinical evidence demonstrates that BTK inhibitors exert dual regulatory effects in CAR‐T therapy for MCL, CLL, and DLBCL: enhancing antitumor efficacy by promoting in vivo CAR‐T cell expansion and significantly reducing CRS severity [188, 219, 279]. Several clinical studies in relapsed/refractory lymphoma are underway (NCT05202782, NCT05744037, NCT04257578, among others) (Table 4).

**TABLE 4:** The ongoing clinical trials of BTK inhibitors combined with CAR‐T cell therapy in B‐cell malignancies.

Type of inhibitor	Clinical trial	Phase	Start year	Patient population	BTKi + CAR‐T strategy	Disease type	BTKi + CAR‐T cell therapy
Covalent	NCT03960840	I/II	2019	CLL/SLL *N* = 224	Ibrutinib + Rapcabtagene Autoleucel	Patients with CLL/SLL demonstrating SD/PR after ≥6 months of ibrutinib in the second‐ or subsequent‐line setting	Dose‐escalation and expansion of rapcabtagene autoleucel combined with ibrutinib
	NCT05020392	III	2021	R/R CD19^+^ B‐cell lymphoma (MCL, FL, BL, CLL, DLBCL) (*N* = 24)	BTKi (ibrutinib, zanubrutinib, or orelabrutinib) + CD19 CAR‐T	Failure of ≥1 prior line of therapy	Oral BTKi initiated at enrollment and continued for up to 90 days (or longer for patients deriving benefit) after CD19 CAR‐T cell infusion.
	NCT05744037	II	2023	R/R B‐NHL *N* = 30	Ibrutinib + CD19 CAR‐T	No effective treatment options available; ineligible for HSCT, or relapsed/refractory after transplantation	N/A
	NCT05672173	II	2023	Richter's syndrome *N* = 20	Ibrutinib + nivolumab + lisocabtagene maraleucel	R/R after ≥2 prior systemic therapies with no prior PD‐1 inhibitor exposure	N/A
	NCT06482684	II	2024	MCL *N* = 150	Ibrutinib + rituximab + brexucabtagene autoleucel	Treatment‐naïve high‐risk MCL	Two cycles of rituximab and ibrutinib before CAR‐T cell therapy, followed by 6 months of ibrutinib maintenance
	NCT04257578	I/II	2020	DLBCL, FL *N* = 20	Acalabrutinib + CD19 CAR‐T (axicabtagene ciloleucel)	Histologically confirmed large B‐cell lymphoma; patients known to be refractory to BTK inhibitors are excluded	Acalabrutinib initiated up to 3 weeks and at least 24 h before leukapheresis, continued until disease progression or unacceptable toxicity
	NCT04484012	II	2020	R/R MCL *N* = 36	Acalabrutinib + anti‐CD19 CAR‐T	Participants received acalabrutinib for 3–7 months; patients refractory to any BTKi were excluded	Initiated 5 days before CAR‐T cell infusion through day 28 postinfusion, followed by 28‐day monthly cycles for up to 6 months
	NCT05256641	I/II	2025	R/R DLBCL *N* = 24	Acalabrutinib + CAR‐T/HSCT	Eligible patients with double‐/triple‐hit lymphoma, secondary CNS involvement, transformed indolent lymphoma, or high‐risk IPI (4–5) before CAR‐T	Acalabrutinib administered as maintenance therapy (orally twice daily) from day 28 to day 104 post‐CAR‐T infusion through 1 year, unless disease progression or unacceptable toxicity occurs
	NCT05202782	II	2022	R/R NHL R/R DLBCL *N* = 24	Zanubrutinib + CD19 CAR‐T (axicabtagene ciloleucel, tisagenlecleucel, or lisocabtagene maraleucel)	R/R to standard therapy	Zanubrutinib administered 7–14 days before CAR‐T infusion through days 1–28 postinfusion; repeated for 12 months in the absence of progression or unacceptable toxicity
	NCT05873712	II	2023	Richter's syndrome *N* = 24	Zanubrutinib+ Lisocabtagene maraleucel	Patients who received ≥1 prior systemic therapy or developed RS while on CLL treatment	Zanubrutinib administered concurrently with lisocabtagene maraleucel
	NCT06695013	II	2024	R/R B‐NHL *N* = 144	Zanubrutinib± radiotherapy + CAR‐T	Patients must have received ≥1 prior line of therapy, including an anthracycline‐based regimen and anti‐CD20 monoclonal antibody	Zanubrutinib ± radiotherapy as bridging therapy; post‐CAR‐T day 28 response‐guided management: CR received no maintenance, PR received zanubrutinib plus a PD‐1 inhibitor for 2 years, and SD/PD were excluded
	NCT07261163	II	2025	R/R DLBCL *N* = 76	Zanubrutinib + CD19 CAR‐T	Relapse within 6 weeks before enrollment; no prior exposure to BTK or PD‐1 inhibitors required for eligibility	Zanubrutinib administered to patients experiencing disease progression after CD19 CAR‐T immunotherapy
	NCT07341191	II	2026	R/R DLBCL *N* = 40	Zanubrutinib+ sonrotoclax + CAR‐T	Participants must have received ≥1 prior systemic therapy including a BTKi; prior BCL2 inhibitor exposure permitted if no progression within 6 months	Zanubrutinib combined with sonrotoclax ramp‐up during induction, followed by CAR‐T therapy and zanubrutinib maintenance
Noncovalent	NCT05990465	I	2025	R/R B‐cell malignancies *N* = 12	Pirtobrutinib + LV20.19 CAR‐T	R/R B‐cell non‐Hodgkin lymphoma, including Richter transformation	Pirtobrutinib administered as bridging therapy starting ≥14 days before leukapheresis, continued until lymphodepletion, and resumed post‐CAR‐T infusion from day 28 to day 120, with maintenance for up to 1 year
	NCT06553872	II	2024	R/R MCL	Pirtobrutinib + brexucabtagene autoleucel	Patients receiving <2 months of maintenance therapy before CAR‐T evaluation without disease progression	Pirtobrutinib initiated 27 days before CAR‐T infusion and continued as maintenance for 1 year postinfusion

Abbreviations: BTKi, Bruton's tyrosine kinase inhibitor; CAR‐T, chimeric antigen receptor T‐cell; R/R, relapsed or refractory; CLL, chronic lymphocytic leukemia; DLBCL, diffuse large B‐cell lymphoma; FL, follicular lymphoma; MCL, mantle cell lymphoma; NHL, non‐Hodgkin lymphoma; B‐NHL, B‐cell non‐Hodgkin lymphoma; BL, Burkitt lymphoma; RS, Richter syndrome; HSCT, hematopoietic stem cell transplantation; N/A, not available.

*Data sources*: ClinicalTrials.gov (clinicaltrials.gov) and PubMed.

In patients with refractory B‐cell lymphoma who failed initial anti‐CD19 CAR‐T therapy, subsequent ibrutinib‐based salvage followed by a second CAR‐T infusion has been associated with enhanced CAR‐T expansion and improved response rates. However, CRS severity increased from grade 0–2 after the first infusion to grade 2–4 after the second, potentially due to higher tumor burden and altered CAR‐T expansion kinetics [274]. Despite this increase, overall toxicity remained manageable and comparable to historical CAR‐T monotherapy, consistent with clinical observations that BTK inhibitor exposure does not substantially exacerbate treatment‐related adverse events. Nevertheless, the intrinsic adverse effects of BTK inhibitors themselves warrant consideration [280]. To date, no evidence suggests that administering BTK inhibitors during lymphodepletion or immediately after CAR‐T cell infusion exacerbates toxicity or diminishes efficacy [281]. Attention should be paid to the intrinsic toxicities of BTK inhibitors, including bleeding risk, infection susceptibility, and potential drug–drug interactions. Particular caution is required with ibrutinib, which has been clearly associated with cardiac arrhythmias, including reported cases of ventricular arrhythmia and sudden death [144, 282, 283]. Therefore, for patients with B‐cell malignancies receiving sequential BTK inhibitor maintenance following CAR‐T therapy, thorough cardiovascular assessment and enhanced electrocardiographic monitoring are recommended.

Preclinical and clinical studies have demonstrated synergistic interactions between CAR‐T cells and BTK inhibitors; however, the optimal combination strategy (sequential versus concurrent), the timing of BTK inhibitor administration (onset and duration), and the specific patient populations that would benefit most remain to be further investigated [284]. Given the manufacturing time of CAR‐T cells (typically 3–6 weeks), treatment delays may lead to disease progression, functional T‐cell decline, and even patient attrition [285], with approximately 7% of patients reported to die during this interval [286]. These challenges underscore the critical role of bridging therapy between leukapheresis and lymphodepletion, particularly for patients with aggressive disease. Importantly, baseline tumor burden is a critical prognostic factor that not only affects the durability of CAR‐T responses but is also positively correlated with CRS severity [287]. Furthermore, prognostic analyses have identified that durable remission is closely associated with deep initial response, low baseline tumor volume, absence of extramedullary disease, and higher peak levels of circulating CAR‐T cells [288].

Emerging clinical evidence suggests that BTK inhibitor‐based bridging therapy not only reduces tumor burden before CAR‐T infusion but also enhances CAR‐T cell expansion and response rates. Concurrently, BTK inhibition may mitigate CRS severity, likely through modulation of the inflammatory milieu, although prolonged exposure may increase hematologic toxicity and higher‐grade CRS in certain settings. Consistently, in patients receiving CD19 CAR‐T therapy, lower tumor burden at infusion was associated with reduced CRS incidence [289], further supporting the rationale for integrating BTK inhibitors as bridging agents in CAR‐T treatment strategies.

Multiple clinical studies have substantiated the synergistic effects of BTK inhibitor‐based bridging strategies in combination with CAR‐T therapy. In a phase I/II study involving R/R CLL patients who had failed prior ibrutinib therapy, patients received ibrutinib for at least 2 weeks before leukapheresis and continued for a minimum of 3 months following CAR‐T cell infusion [188]. The combination therapy group exhibited a higher ORR than CAR‐T monotherapy, effectively preventing disease progression due to ibrutinib discontinuation and reducing CRS severity. Mechanistically, ibrutinib exposure at the time of leukapheresis was associated with enhanced ex vivo T‐cell expansion and increased proportions of central memory CD4^+^ and CD8^+^ T cells [275], which may contribute to improved CAR‐T efficacy in high‐risk CLL [257].

In MCL, real‐world data further reinforce these observations. In a retrospective cohort of 95 patients with R/R MCL, predominantly BTKi‐exposed (98%), including a high proportion of refractory cases (76%), brexucabtagene autoleucel achieved high response rates (ORR 89%, CR 81%) with durable outcomes (median PFS 16.4 months; 12‐month OS 75%). A subset of patients received BTKi‐based bridging (31%) or BTKi combined with venetoclax (14%), without apparent compromise in efficacy [290]. Consistent findings were observed in another real‐world cohort (*N* = 66), in which all patients had prior BTKi exposure and 56% were refractory. CAR‐T therapy achieved an ORR of 86% (CR 77%), with 6‐month PFS and OS rates of 77% and 88%, respectively. Notably, BTKi‐based bridging (alone or combined with venetoclax) was administered in a subset of patients, again supporting the feasibility of this strategy in heavily pretreated populations [291]. Although CRS was common across studies (approximately 80%–90%), the incidence of severe CRS (grade ≥3) remained relatively low (8%–10%), and toxicities, including neurologic events and cytopenias, were generally manageable.

In a retrospective study of 17 patients with R/R DLBCL, all received zanubrutinib for ≥2 months (median 4 months) before sequential CAR‐T therapy. After a median follow‐up of 30 months, the best ORR was 88.2% with a CR rate of 70.5%. The 24‐month PFS and OS rates were 59% and 71%, respectively, and the 36‐month OS rate reached 65%. Although CRS occurred in 11 patients, all cases were effectively managed. Subgroup analysis showed no significant OS difference between patients with high‐risk features (bulky disease, elevated lactate dehydrogenase [LDH], extranodal involvement, or TP53 mutations) and those without, suggesting that zanubrutinib may mitigate the adverse prognostic impact of these factors [292]. The duration of BTK inhibitor exposure also appears to influence outcomes. A recent study evaluating varying durations of BTK inhibitor bridging before anti‐CD19 CAR‐T therapy found that prolonged bridging (≥2 months) correlated with higher ORR, enhanced CAR‐T expansion, reduced PD‐1 expression, and increased activation‐related gene signatures. However, extended exposure may increase the incidence of grade ≥2 CRS and hematologic toxicities, while ICANS rates remained largely unaffected [293]. This paradox of improved CAR‐T fitness alongside increased toxicity likely reflects the ability of BTKi bridging to remodel the pre‐leukapheresis T‐cell repertoire, generating a more vigorous effector population whose robust initial activation drives a heightened inflammatory response. Collectively, these data support BTK inhibitors as effective bridging agents that enhance CAR‐T fitness and antitumor activity, though efficacy gains must be balanced against potential increases in immune‐related toxicity. Notably, PFS and OS did not differ significantly between CAR‐T monotherapy and CAR‐T combined with autologous hematopoietic stem cell transplantation (HSCT) [292], whereas ibrutinib may offer additional advantages as a bridging strategy before HSCT [188].

Updated clinical guidelines endorse combining BTK inhibitors with CAR‐T cell therapy. The 2025 CSCO guidelines recommend BTK inhibitors as bridging therapy to control high disease burden and optimize patient status before CAR‐T infusion. For patients with R/R MCL previously treated with covalent BTK inhibitors, CAR‐T therapy is an appropriate subsequent option upon disease progression, and early implementation may be warranted in those with high‐risk features [281]. Concurrent administration of BTK inhibitors and CAR‐T therapy has also been explored; although not yet approved, this approach may benefit select populations. However, potential drug interactions and immunotoxicities (particularly the increased risk of CRS and neurotoxicity with ibrutinib) require careful monitoring.

Current evidence indicates that rational integration of BTK inhibitors into the CAR‐T treatment cycle (before or after infusion) can broaden the therapeutic window for B‐cell malignancies by improving both efficacy and safety. Ibrutinib currently has the most robust supporting data, with mechanisms that include enhancing T‐cell function, modulating the tumor microenvironment, and mitigating CRS risk. Despite this promise, key questions regarding optimal sequencing, patient selection, and long‐term safety remain unresolved and require well‐designed multicenter trials. Ongoing global registration studies are expected to provide high‐level evidence to guide precise clinical implementation.

#### 4.3.2 BTK Inhibitors and Immune Checkpoint Inhibitors

Several clinical studies have evaluated the combination of BTK inhibitors with ICIs in B‐cell malignancies [163]. A single‐arm phase II trial of ibrutinib plus nivolumab in 17 adults with relapsed/refractory classical Hodgkin lymphoma (R/R cHL) reported an ORR of 51.9% (CRR, 29.4%) [294]. Notably, among 10 patients who had progressed on prior nivolumab monotherapy, the ORR and CRR were 50% and 20%, respectively, suggesting that antitumor immunity may be restored despite previous ICI failure. Prior PD‐1 inhibitor exposure did not significantly affect PFS, supporting an immunomodulatory role for ibrutinib in overcoming immune escape. In a phase I/II study of ibrutinib plus nivolumab in R/R NHL and CLL, the combination demonstrated acceptable tolerability, with diarrhea, neutropenia, and fatigue as the most common adverse events [295]. Grade 3–4 events were primarily neutropenia and anemia, and serious adverse events included anemia and pneumonia. Regarding efficacy, response rates in CLL, FL, and DLBCL were comparable to those reported for ibrutinib monotherapy, whereas higher response rates were observed in the Richter transformation (RT) subtype, suggesting potential benefit in this biologically aggressive disease. Consistent findings were reported in a separate trial evaluating ibrutinib combined with the PD‐L1 inhibitor durvalumab in patients with R/R FL and DLBCL [296]. The ORRs were 26% in FL, 13% in GCB DLBCL, and 38% in non‐GCB DLBCL, with a median PFS of 4.6 months and a median OS of 18.1 months. Mechanistically, non‐GCB DLBCL relies on chronically active BCR signaling and is sensitive to BTK inhibition; NF‐κB activation may further render this subtype more responsive to combined BTK inhibition and PD‐L1 blockade [203, 296]. Nevertheless, the study also reported limited overall efficacy and a high incidence of durvalumab‐related adverse events, including neutropenia, fatigue, and rash [296]. In another study, the second‐generation BTK inhibitor zanubrutinib was combined with the PD‐1 inhibitor tislelizumab in patients with R/R B‐cell lymphomas [297]. Although early data indicated some antitumor activity and a manageable toxicity profile, efficacy data specific to DLBCL or FL subgroups remain lacking. Overall, despite a strong biological rationale and preclinical support for combining BTK inhibitors with ICIs, clinical trials to date have not demonstrated a significant advantage over BTK inhibitor monotherapy, and increased toxicity coupled with limited incremental benefit has constrained their clinical development.

RT represents a highly unfavorable form of CLL progression characterized by the development of aggressive lymphoma [298]. Patients treated with chemoimmunotherapy regimens such as R‐CHOP typically achieve an ORR below 40% and a median OS of only 6–8 months [299]. Monotherapies including ICIs, BTK inhibitors, and BCL‐2 inhibitors have shown efficacy in select cases [300, 301]. A prospective, multicenter phase II trial evaluated zanubrutinib combined with the PD‐1 inhibitor tislelizumab in patients with RT [302]. Among 48 patients, the ORR was 58.3%, with a CR rate of 18.8% and a PR rate of 39.6%. The median PFS was 10 months, and the 12‐month PFS rate was 46.9%. Common adverse events included infections, gastrointestinal symptoms, and hematologic toxicities. The combination demonstrated favorable tolerability and durable responses, representing a promising immuno‐targeted strategy for RT.

In recent years, combining BTK inhibitors with ICIs has attracted growing interest in PCNSL. Prior studies have demonstrated the potential efficacy of BTK inhibitors in this setting, particularly in patients with CD79B and MYD88 mutations, who exhibited high response rates [172, 303]. However, the efficacy of ibrutinib monotherapy in R/R PCNSL remains limited, underscoring the need for improved therapeutic strategies [304].

ICIs have been applied across various tumor types to prolong survival, and anti‐PD‐1 monoclonal antibodies have shown notable activity in R/R PCNSL [305]. In one case report, a CD20‐negative PCNSL patient who had failed methotrexate‐based therapy achieved a CR after only two cycles of zanubrutinib plus tislelizumab and remained in remission for 20 months, suggesting potential synergistic effects of this combination [213]. Preliminary results from a phase II study of orelabrutinib combined with the PD‐1 antibody sintilimab in R/R PCNSL also demonstrated promising efficacy [306]. Patients receiving daily orelabrutinib and triweekly sintilimab experienced no severe immune‐related toxicities. The ORR reached 61.5%, comprising 4 CRs, 1 near‐complete response (CRu), and 3 PRs, further supporting the therapeutic potential of BTK inhibitor–ICI combinations in PCNSL. Several ongoing clinical trials are evaluating such combinations, including acalabrutinib plus durvalumab (NCT04462328) and ibrutinib plus nivolumab (NCT03770416).

Beyond blocking the BCR signaling pathway, BTK inhibitors suppress the activation of NFATc1 and STAT3, leading to downregulation of PD‐L1 expression and indirectly modulating tumor immune evasion [202, 203]. Additionally, as BTK is a key downstream mediator of the CXCR4–CXCL12 axis, its inhibition may enhance the efficacy of anti‐PD‐1/PD‐L1 monoclonal antibodies by interfering with CXCR4 signaling. These immunomodulatory effects provide a strong rationale for combining BTK inhibitors with ICIs, a strategy that has demonstrated synergistic antitumor activity in preclinical models and preliminary clinical studies [200]. Across various B‐cell lymphomas, particularly in relapsed/refractory cases and molecularly defined subtypes, this combination has shown promising clinical potential.

In summary, current preclinical and limited clinical evidence supports the feasibility of combining BTK inhibitors with ICIs. This approach warrants further investigation, particularly in patients with immunosuppressive TMEs or T‐cell exhaustion following CAR‐T cell therapy. Large‐scale, mechanism‐driven clinical trials will be essential to confirm efficacy and safety and to expand the clinical utility of this strategy in cancer immunotherapy.

#### 4.3.3 BTK Inhibitors and CD20‐Targeted Antibodies

The combination of BTK inhibitors with anti‐CD20 monoclonal antibodies (such as rituximab and obinutuzumab) was among the earliest targeted regimens to enter clinical practice and remains widely used in CLL/SLL, WM, and selected indolent B‐cell lymphomas. Mechanistically, BTK inhibition disrupts CXCR4‐ and CXCR5‐mediated chemotaxis and integrin‐dependent adhesion, mobilizing CLL cells from lymphoid tissues into the peripheral blood [254, 307]. Clinically, the addition of rituximab or obinutuzumab accelerates lymph node shrinkage and promotes faster resolution of treatment‐related lymphocytosis, reflecting improved early disease kinetics [308, 309].

Ibrutinib combined with glycoengineered anti‐CD20 antibodies or ofatumumab has achieved high response rates in patients with relapsed/refractory CLL, with an overall response rate (ORR) of 83% [310, 311]. In previously untreated high‐risk patients harboring del(17p), del(11q), TP53 mutation, or unmutated IGHV, the combination of ibrutinib and obinutuzumab significantly prolonged PFS, with a 3‐year PFS rate of 79% [308]. On the basis of these results, the ibrutinib–obinutuzumab regimen was approved in 2019 for treatment‐naïve CLL. Second‐generation BTK inhibitors (acalabrutinib and zanubrutinib) combined with anti‐CD20 antibodies have also demonstrated favorable tolerability and durable efficacy [309]. In Waldenström macroglobulinemia, recent pooled analyses further suggest that the benefit of combining ibrutinib with rituximab may be biomarker‐dependent, with the most pronounced advantage observed in patients harboring CXCR4 mutations [312].

Despite these benefits, deep remissions remain uncommon with BTKi‐based doublet therapy. The phase III Alliance A041202 trial showed that ibrutinib plus rituximab did not improve PFS compared with ibrutinib monotherapy, although initial disease clearance was faster [313]. Similarly, MRD negativity rates remain low with BTKi‐anti‐CD20 combinations, underscoring the limited capacity of continuous BTK inhibition to eradicate residual disease. The depth of response may also depend on antibody selection, as obinutuzumab‐based regimens have been associated with higher complete response rates than rituximab‐based combinations [307].

Current research has shifted toward fixed‐duration, BTKi‐centered triplet regimens, such as BTK inhibitors combined with venetoclax and obinutuzumab, which achieve higher undetectable MRD rates and superior PFS compared with chemoimmunotherapy, supporting chemotherapy‐free strategies [314]. Pirtobrutinib is also being explored in frontline settings. An ongoing phase II trial (NCT06333262) is evaluating fixed‐duration pirtobrutinib plus obinutuzumab in previously untreated CLL. Additionally, a time‐limited triplet regimen of pirtobrutinib, venetoclax, and obinutuzumab has shown promising activity, achieving undetectable MRD at a sensitivity of 10^−^^6^ in 80%–85% of patients at 12 months. The safety profile is consistent with that of the individual agents, and longer follow‐up is ongoing to assess the durability of response [315].

Parallel to these developments, interest has grown in combining BTK inhibitors with CD20×CD3 bispecific antibodies (BsAbs), which redirect endogenous T cells to eliminate malignant B cells. BTK inhibition may enhance BsAb efficacy by modulating the TME and improving T‐cell functional fitness, thereby facilitating more effective immune‐mediated cytotoxicity. Early clinical data support this concept. Functional studies using primary CLL samples demonstrated that the CD3×CD20 BsAb epcoritamab induces autologous T‐cell‐mediated cytotoxicity in an effector‐to‐target ratio‐dependent manner, independent of CD20 surface expression on CLL cells. Notably, epcoritamab activity was further enhanced in the context of ongoing BTKi therapy or in combination with the BCL‐2 inhibitor venetoclax and was associated with reduced tumor burden in peripheral blood and spleen in patient‐derived xenograft models. These findings support BTK inhibitors as a rational immunomodulatory partner to sensitize and consolidate responses to CD20‐directed BsAb therapy [201]. Consistent with this, an immune‐profiling study of ibrutinib‐based therapy reported reinvigoration of CD8^+^ T‐cell function and demonstrated that such treatment states could be exploited using the CD20×CD3 BsAb glofitamab in functional assays, motivating combination immunotherapy strategies [316]. Clinically, early‐phase combination data are now emerging in indolent lymphoma. In a 2025 report of mosunetuzumab (CD20×CD3) plus zanubrutinib for high‐tumor‐burden FL (including untreated and relapsed/refractory patients), the regimen produced an ORR of 92% with 46% complete responses at the data cutoff. CRS occurred in 65% of patients but was predominantly grade 1–2, suggesting that BTKi–BsAb co‐administration is feasible with manageable acute immune toxicity under appropriate protocols [316]. In MCL, a phase II study evaluating glofitamab in combination with the noncovalent BTKi pirtobrutinib, incorporating obinutuzumab pretreatment and step‐up dosing of glofitamab, is assessing endpoints including complete response and MRD negativity, reflecting a therapeutic strategy aimed at achieving deep remissions through a fixed‐duration approach with MRD‐guided treatment discontinuation (NCT05833763).

Collectively, these findings indicate that while BTK inhibitors combined with CD20 monoclonal antibodies remain an important therapeutic backbone for accelerating early disease control, the field is increasingly transitioning toward more complex BTKi‐centered combinations, including venetoclax‐based triplets and BTKi–BsAb regimens, aimed at achieving deeper responses, MRD eradication, and ultimately time‐limited treatment strategies in B‐cell malignancies.

#### 4.3.4 BTK Inhibitors and BCL2 Inhibitors

In B‐cell lymphomas, upregulation of BCL‐2 as part of pro‐survival signaling contributes to resistance to BTK inhibitors, suggesting that BCL‐2 inhibition may restore sensitivity to BTK‐targeted therapy [317, 318]. The synergy between BTK and BCL‐2 inhibitors is primarily mediated through reprogramming of the anti‐apoptotic network. By suppressing BCR‐NF‐κB signaling and disrupting microenvironment‐derived survival cues, BTK inhibition downregulates alternative anti‐apoptotic proteins such as MCL‐1 and BCL‐XL, enhances mitochondrial priming, and shifts malignant cells toward increased BCL‐2 dependence. Additionally, BTK inhibitors impair CXCR4/CXCL12‐ and integrin‐mediated stromal interactions, mobilizing tumor cells from protective niches into the circulation [319, 320, 321]. This complementary targeting reduces proliferation and microenvironmental support, while venetoclax efficiently eliminates quiescent circulating cells, enabling more comprehensive tumor clearance and providing a biological basis for MRD eradication [322]. In ibrutinib‐resistant DLBCL, TLR‐driven NF‐κB activation may increase BCL‐2 expression, further supporting the rationale for combined BCL‐2 blockade [323]. Preclinical studies consistently demonstrate superior antitumor activity of the combination compared with either agent alone.

Multiple clinical studies have evaluated the combination of ibrutinib and venetoclax in B‐cell malignancies. Clinical evidence supports this regimen as an effective chemotherapy‐free strategy capable of inducing deep and durable remissions in CLL/SLL. Early phase II studies reported high overall response rates and uMRD, with a PFS rate of 93% and bone marrow uMRD achieved in 66% of patients [324]. The MRD‐guided cohort of the CAPTIVATE trial confirmed high uMRD rates in both peripheral blood and bone marrow, with sustained disease control after treatment discontinuation. Fixed‐duration regimens have demonstrated durable PFS with manageable toxicity, including in patients with comorbidities [325]. Phase III evidence from the GLOW trial and long‐term follow‐up analyses further showed that fixed‐duration ibrutinib–venetoclax significantly improved PFS compared with chemoimmunotherapy, leading to regulatory approval in Europe for CLL [326].

Beyond CLL, this combination has shown activity in other B‐cell malignancies, including DLBCL. In a phase II study enrolling relapsed/refractory and untreated DLBCL patients, 71% achieved complete remission and 67% achieved MRD negativity, outcomes superior to historical results with monotherapy [327]. Collectively, these findings support BTK inhibitor plus venetoclax as a highly effective targeted strategy capable of achieving deep responses and potential functional disease control. Ongoing trials are evaluating next‐generation BTK inhibitors in combination with venetoclax, including zanubrutinib (NCT05168930) and pirtobrutinib (NCT05734495), in hematologic malignancies [155].

#### 4.3.5 BTK Inhibitors and Hematopoietic Stem Cell Transplantation

In the setting of HSCT, BTK inhibitors have demonstrated clinical value in both disease control and posttransplant immune modulation. Mechanistically, BTK signaling regulates B‐cell activation, antigen presentation, and cytokine production, all of which contribute to the pathogenesis of chronic graft‐versus‐host disease (cGVHD) [328]. In patients with relapsed/refractory B‐cell malignancies undergoing allogeneic HSCT, pretransplant BTK inhibitor therapy, particularly ibrutinib, can serve as a bridging strategy to reduce tumor burden and improve MRD status, thereby optimizing disease control at transplantation and potentially lowering relapse risk [329].

Clinical observations further indicate that ibrutinib retains antitumor activity with acceptable tolerability in CLL patients who relapse after HSCT, supporting its role as posttransplant maintenance or salvage therapy [330]. The TRIANGLE trial (NCT02858258) demonstrated that adding ibrutinib to standard chemoimmunotherapy followed by ASCT and ibrutinib maintenance significantly improved 3‐year failure‐free survival to approximately 88% in transplant‐eligible MCL patients, compared with standard chemotherapy plus ASCT (failure‐free survival ∼72%), supporting the integration of BTK inhibition into frontline management strategies.

Beyond their direct antitumor effects, BTK inhibitors exert immunomodulatory activity by simultaneously inhibiting BTK in B cells and ITK in T cells, thereby reducing Th2 and Th17 responses and attenuating profibrotic and inflammatory pathways associated with cGVHD [331]. This provides a mechanistic rationale for their use in GVHD management. Preclinical studies further support a preventive role, showing that dual BTK/ITK inhibition alleviates both acute and chronic GVHD and improves survival [329, 332]. In a prospective multicenter study of steroid‐refractory cGVHD, ibrutinib achieved a best overall response rate of approximately 67%, with durable responses lasting ≥20 weeks and a significant reduction in corticosteroid requirements [333]. Correlative analyses demonstrated decreased inflammatory and profibrotic mediators and clinical activity across multiple organ systems, suggesting restoration of posttransplant immune balance [334]. These findings led to FDA approval of ibrutinib for patients with cGVHD after failure of at least one prior systemic therapy.

The potential roles of BTK inhibitors in the HSCT setting include pretransplant bridging to optimize disease status, posttransplant maintenance or relapse management, and treatment or prevention of GVHD. Prospective randomized studies are needed to define the optimal timing, duration, and combination strategies while balancing relapse control against the risks of infection and excessive immunosuppression.

### 4.4 Emerging Roles of BTK Inhibitors in Solid Tumors

BTK is a central mediator of BCR signaling that functions as both an oncogenic driver and a therapeutic target. Recent studies have extended the oncogenic role of BTK beyond hematologic malignancies to various solid tumors. Aberrant expression of BTK isoforms, particularly the oncogenic p65BTK variant, has been identified in colorectal, ovarian, prostate, and breast cancers, as well as in a subset of non–small cell lung cancers (NSCLC). In these malignancies, elevated p65BTK expression is closely associated with enhanced tumor cell survival, underscoring its critical role in solid tumor biology [335, 336, 337, 338].

Unlike canonical BTK expressed in hematopoietic cells, p65BTK acts as a potent oncogenic driver by interacting with key signaling pathways such as RAS/MAPK and ERK1/2, thereby promoting tumor cell proliferation, survival, and chemoresistance [339, 340]. Furthermore, BTK overexpression is associated with cytoskeletal remodeling, increased expression of genes involved in cell adhesion and extracellular matrix organization, and enhanced tumor invasiveness, all of which correlate with poor clinical outcomes [341].

BTK inhibitors such as ibrutinib suppress tumor cell proliferation, induce apoptosis, and enhance chemosensitivity while simultaneously remodeling the TME by reducing immunosuppression and promoting antitumor immunity. These effects provide a strong mechanistic basis for repurposing BTK inhibitors in solid tumors and integrating them into combination immunotherapy strategies [14, 15, 342].

In ovarian cancer, in vitro studies demonstrate that elevated BTK expression and enrichment of cancer stem cell (CSC) markers are associated with cisplatin resistance. Ibrutinib exhibits synergistic activity when combined with cisplatin, suggesting potential utility as a platinum sensitizer. Mechanistically, BTK promotes chemoresistance through activation of the JAK2/STAT3 pathway, upregulating downstream effectors such as SRY‐box transcription factor 2 (SOX2) and BCL‐XL to sustain CSC self‐renewal and survival [343]. Early translational studies and clinical observations further support this potential, reporting reductions in CA‐125 levels in platinum‐resistant cases [344].

In breast cancer, particularly HER2‐positive and triple‐negative subtypes, aberrant expression of BTK and TEC family kinases is closely associated with tumor progression. Ibrutinib induces apoptosis in HER2‐overexpressing cells via a caspase‐dependent extrinsic pathway and promotes cell‐cycle arrest through modulation of STAT3 (Y705) and p21 (T145) phosphorylation [345]. Additionally, BTK inhibition enhances antitumor immunity by synergizing with PD‐L1 blockade and promoting differentiation of MDSCs into mature dendritic cells [200, 346]. Combination strategies with other targeted agents, such as FTO inhibitors, further suppress oncogenic pathways including c‐Myc and E2F1 [347].

In pancreatic cancer, BTK inhibitors have demonstrated antiproliferative and antifibrotic effects in preclinical models and show favorable tolerability when combined with gemcitabine [348]. Ibrutinib also acts as a radiosensitizer by inhibiting EGFR and AKT/mTOR signaling pathways, thereby inducing G2/M cell cycle arrest and promoting apoptosis to enhance radiotherapy efficacy [349].

In colorectal cancer, p65BTK overexpression is associated with ERK1/2 activation and poor prognosis, particularly in advanced‐stage disease [340, 350]. BTK inhibitors exhibit dose‐dependent cytotoxicity, and targeting p65BTK reverses 5‐fluorouracil resistance in drug‐resistant colorectal cancer cells, organoids, and in vivo models [340, 351]. Moreover, in CT26 xenograft models, combination therapy with ibrutinib and PD‐L1 blockade induces complete tumor regression and establishes durable immune memory [200]. Similarly, in NSCLC, head and neck squamous cell carcinoma (HNSCC), and glioma, BTK or its isoforms promote tumor progression through pathways such as RAS/MAPK, NF‐κB, and EGFR, and their inhibition suppresses proliferation and induces apoptosis [339, 340, 352, 353].

Despite compelling preclinical evidence, clinical data remain limited and largely exploratory, with most studies focusing on combination strategies. For example, a phase I/II trial of ibrutinib combined with the PD‐L1 inhibitor durvalumab demonstrated acceptable safety but limited efficacy in solid tumors [354]. In breast cancer, trials combining ibrutinib with trastuzumab (NCT03379428) are ongoing, whereas the phase III RESOLVE trial in pancreatic cancer (NCT02436668) failed to improve overall survival and showed inferior progression‐free survival, indicating that this approach cannot be recommended [355]. Studies in colorectal cancer combining ibrutinib with pembrolizumab have similarly demonstrated limited clinical benefit [356].

These largely negative findings highlight a critical disconnect between preclinical rationale and clinical efficacy attributable to several nonmutually exclusive factors. First, unlike in B‐cell malignancies where BTK serves as a central oncogenic driver, BTK signaling in solid tumors is often context‐dependent and predominantly confined to tumor‐infiltrating immune cells rather than tumor cells themselves; consequently, its inhibition may not sufficiently disrupt core tumor survival pathways. Second, pharmacokinetic and pharmacodynamic limitations may further attenuate clinical activity, including suboptimal intratumoral drug penetration, incomplete or heterogeneous BTK occupancy within the TME, and discrepancies between peripheral and tissue‐level target engagement. Third, the complexity and redundancy of the TME likely blunt the impact of BTK inhibition. Although preclinical models suggest that BTK blockade can reprogram myeloid cells and enhance antitumor immunity, these effects may be insufficient to overcome dominant immunosuppressive pathways such as T‐cell exhaustion, stromal‐mediated barriers, and alternative checkpoint signaling in advanced solid tumors.

Collectively, these findings indicate that BTK inhibition alone is insufficient as a primary therapeutic strategy in solid tumors. The discrepancy between preclinical efficacy and clinical outcomes likely reflects pharmacokinetic limitations, TME complexity, and the nondominant role of BTK as an oncogenic driver in these cancers. Future studies should therefore focus on leveraging BTK inhibitors as chemosensitizers or immunomodulatory agents within rational combination strategies.

## 5 BTK Inhibitors in Nonmalignant Diseases

Beyond their established role in B‐cell malignancies, BTK inhibitors have demonstrated efficacy in autoimmune and inflammatory disorders, underscoring the central role of BTK in both adaptive and innate immune regulation. Autoreactive B cells contribute to disease progression and sustain chronic immune activation through MHC‐II‐mediated antigen presentation, co‐stimulation of pathogenic T cells (e.g., via CD80/CD86), differentiation into antibody‐secreting plasma cells, and production of pro‐inflammatory cytokines such as IL‐6 [357, 358]. Although the first‐in‐class inhibitor ibrutinib validated the feasibility of targeting this pathway, its off‐target kinase inhibition and associated toxicities limit long‐term use in nonmalignant conditions [359]. These limitations have driven the development of next‐generation, more selective BTK inhibitors with improved safety profiles suitable for chronic autoimmune diseases.

### 5.1 Immune Thrombocytopenia

Immune thrombocytopenia (ITP) is an autoimmune disorder characterized by peripheral platelet destruction and impaired bone marrow platelet production [113, 360]. In adults, first‐line therapies typically aim to reduce platelet destruction to minimize bleeding and rapidly increase platelet counts, although durable long‐term responses are limited. Mechanistic studies have shown that IgG autoantibodies bind platelet glycoprotein antigens on megakaryocytes to promote apoptosis [361] and mediate platelet clearance via Fcγ receptors on splenic and hepatic macrophages, as well as Ashwell–Morell receptors on hepatocytes [362]. BTK is integral to Fcγ receptor‐mediated macrophage function and regulates autoantibody production. Although ibrutinib treatment in patients with B‐cell malignancies and concomitant ITP has achieved high remission rates [363], its inhibition of platelet aggregation due to BTK expression in platelets contraindicates its use in ITP [360]. Conversely, the highly selective BTK inhibitor rilzabrutinib does not affect collagen‐induced platelet aggregation or induce bruising or platelet count fluctuations in healthy volunteers. This oral, reversible agent demonstrated rapid and sustained platelet responses, improved fatigue, and favorable safety in phase III studies of persistent or chronic ITP, becoming the first approved BTK inhibitor for this indication [114]. Additionally, orelabrutinib significantly increased platelet counts in active ITP mouse models by inhibiting plasma cell differentiation, autoantibody production, pro‐inflammatory cytokine secretion, and hepatic and splenic platelet phagocytosis, representing a potential therapeutic option [364]. Zanubrutinib ameliorates refractory ITP by shifting the Fcγ receptor balance toward FcγRIIb [365], and a phase II study confirmed its efficacy and safety in patients with corticosteroid‐refractory or relapsed disease [366].

### 5.2 Systemic Lupus Erythematosus

Systemic lupus erythematosus (SLE) is a multiorgan, multifactorial autoimmune disease characterized by loss of immune tolerance and excessive autoantibody production against self‐antigens, leading to immune complex formation, inflammation, and tissue damage across multiple organs. In SLE patients, BTK expression and activation are elevated, promoting the survival and activation of autoreactive B cells, plasma cell differentiation, and immune complex‐mediated activation of myeloid cells through Fc and Toll‐like receptors [367, 368]. In murine models, BTK inhibition significantly reduces infiltration and accumulation of T cells, B cells, and macrophages; decreases autoantibody production and germinal center responses; and attenuates renal injury [367, 369], indicating potential therapeutic benefits. Consistently, the covalent BTK inhibitor evobrutinib has shown efficacy in mouse models of SLE and rheumatoid arthritis [370], with in vivo studies confirming potent, highly selective BTK inhibition and high target occupancy [371].

Despite this compelling preclinical rationale, the clinical translation of BTK inhibitors in SLE has proven unexpectedly challenging. In a phase II trial of evobrutinib, the drug failed to demonstrate clinically meaningful efficacy despite achieving high target occupancy, suggesting that BTK inhibition alone may not sufficiently address key pathogenic mechanisms in SLE [366]. Similarly, in another phase II study (NCT02908100) involving patients with moderate‐to‐severe active SLE, fenebrutinib demonstrated potent pharmacodynamic effects, significantly reducing phosphorylated BTK and CD19+ B‐cell levels; nonetheless, it failed to meet the primary endpoint of SRI‐4 response at week 24 [372].

These findings underscore the biological heterogeneity and pathway redundancy that characterize SLE pathogenesis. Unlike B‐cell malignancies, where BTK often functions as a dominant oncogenic driver, the inflammatory network in SLE is sustained by multiple parallel and partially independent pathways, including type I interferon signaling, T cell‐mediated help, and long‐lived plasma cells that are largely BTK‐independent. Consequently, although BTK inhibitors can effectively attenuate B‐cell activation and reduce autoantibody levels, these effects may not translate into substantial clinical improvement in unselected patient populations. This disconnect suggests that future therapeutic strategies should move beyond monotherapy, either by identifying biomarker‐defined, B‐cell‐driven subgroups more likely to respond, or by adopting rational combination approaches. For example, dual inhibition strategies, such as combining the BTK inhibitor elsubrutinib with the JAK1 inhibitor upadacitinib, aim to achieve broader suppression of the autoimmune network.

Encouragingly, emerging clinical data suggest that such refined approaches may yield improved outcomes. Orelabrutinib reduced anti‐double‐stranded DNA IgG levels in SLE patients and was well tolerated in mild‐to‐moderate cases [373]. A separate phase II study evaluated the covalent irreversible BTK inhibitor elsubrutinib, alone or combined with the selective JAK1 inhibitor upadacitinib (ABT‐494), in patients with mild‐to‐severe active SLE, showing significant improvements in disease activity, reduced total flares, and prolonged time to first flare [374]. Building on these findings, multiple ongoing clinical trials are evaluating evobrutinib, fenebrutinib, branebrutinib, and elsubrutinib as monotherapy or in combination for efficacy and safety in SLE patients.

### 5.3 Rheumatoid Arthritis

Rheumatoid arthritis (RA) is a chronic systemic autoimmune disease characterized by synovial inflammation, progressive cartilage destruction, and bone erosion [29]. Its pathogenesis involves complex interactions between innate and adaptive immune responses. Jansson et al. [375] identified an X‐linked gene associated with arthritis susceptibility, and XID mice carrying BTK mutations exhibited reduced arthritis incidence, indicating a protective effect of BTK deficiency. These findings have driven the development and evaluation of multiple BTK inhibitors for RA.

BTK inhibitors block B‐cell proliferation and reduce autoantibody levels. In collagen‐induced arthritis mouse models, they suppress FcγRIII‐mediated production of TNF‐α, IL‐1β, and IL‐6 [60, 376]. Various BTK inhibitors, including CGI1746, GDC‐0834, poseltinib, fenebrutinib, and branebrutinib, have demonstrated efficacy in reducing joint inflammation and bone damage in collagen‐ and adjuvant‐induced arthritis models [60, 376, 377, 378]. Early clinical studies support this therapeutic strategy. In a phase IIa trial, spebrutinib significantly decreased serum CXCL13 and MIP‐1β levels, correlating with improved ACR responses [379]. In a randomized phase II study of methotrexate nonresponders, high‐dose fenebrutinib exhibited efficacy comparable to adalimumab, with evidence of targeting both B‐cell and myeloid pathways [378].

The highly potent and selective tricyclic BTK inhibitor SOMCL‐17‐016 dually inhibits B‐cell function and osteoclastogenesis, making it a promising RA candidate [380]. Evobrutinib, a covalent BTK inhibitor capable of penetrating the central nervous system, has demonstrated favorable tolerability in studies of SLE, RA, and multiple sclerosis [381]. Among other BTK inhibitors under investigation (acalabrutinib, NCT02387762; elsubrutinib, NCT03682705; branebrutinib, NCT04186871), BMS‐986142 (NCT02638948) enhanced standard therapy efficacy in RA experimental models [382] but failed to confer additional clinical benefit in patients with moderate‐to‐severe RA and inadequate methotrexate response, underscoring the need for refined strategies [383].

### 5.4 Multiple Sclerosis

Multiple sclerosis (MS) is an inflammatory demyelinating disease of the central nervous system (CNS) caused by abnormal immune activity, leading to delayed nerve conduction and functional deficits [384]. In vitro studies have shown that BTK inhibitors suppress the activation, differentiation, and cytokine release of pro‐inflammatory M1 macrophages. BTK inhibition also reduces mitochondrial respiration in B cells, decreases activation‐induced co‐stimulatory molecule expression, and mediates an anti‐inflammatory shift in B‐cell responses. In experimental autoimmune encephalomyelitis (EAE) models, BTK inhibitors exhibit therapeutic efficacy independent of B‐cell activity [385, 386].

These findings suggest that BTK blockade may represent a therapeutic strategy for MS and could enhance the efficacy of anti‐CD20 therapies such as rituximab [387, 388]. Certain BTK inhibitors penetrate the blood–brain barrier (BBB) and suppress CNS‐intrinsic autoreactive B cells and microglial activation [389, 390]. Tolebrutinib, an irreversible BTK inhibitor with high BBB penetration that binds to the C481 site, led to a dose‐dependent reduction in new gadolinium‐enhancing lesions after 12 weeks of treatment in a phase II trial of patients with MS, with good tolerability and no treatment‐related discontinuations [387]. Fenebrutinib, a noncovalent, reversible BTK inhibitor, demonstrated good tolerability in a phase II trial of patients with relapsing MS and achieved early, potent, and sustained inhibition of new brain lesions [389]. Evobrutinib at 75 mg once daily reduced the number of MRI‐enhancing lesions in patients with relapsing MS but did not affect annualized relapse rates or disability progression, and some patients discontinued due to hepatotoxicity [391]. Larger‐scale studies are needed to fully assess the efficacy and safety of BTK inhibitors in MS, and multiple ongoing clinical trials are evaluating whether BTK inhibition can serve as an alternative or adjunct to anti‐CD20 therapies.

### 5.5 Sjögren's Syndrome

Primary Sjögren's syndrome (pSS) is characterized by B‐cell hyperactivation, autoantibody production (anti‐SSA/Ro and anti‐SSB/La), and lymphocytic infiltration of the salivary and lacrimal glands. The BTK signaling pathway promotes B‐cell activation, differentiation, and autoantibody secretion. Innate immune cells such as dendritic cells further amplify glandular inflammation through TLR‐BTK‐dependent production of cytokines and chemokines, facilitating immune cell recruitment to exocrine tissues [24, 392]. These mechanisms strongly support BTK inhibition as a strategy to target both adaptive and innate immune pathways in pSS.

Several BTK inhibitors have been evaluated clinically, including the noncovalent inhibitor BMS‐986142 and the covalent inhibitors branebrutinib, remibrutinib, and tirabrutinib. Clinical outcomes to date, however, have been disappointing: randomized trials showed that branebrutinib (NCT04186871) and tirabrutinib (NCT03100942) failed to demonstrate significant clinical benefit [393]. The BMS‐986142 monotherapy trial (NCT02843659) was terminated for failing to meet its primary endpoint, and clinical data for remibrutinib (NCT04035668) remain limited. These findings suggest that, despite strong biological plausibility, BTK inhibition alone may be insufficient to reverse established glandular damage. Future work should better define the role of B cells and BTK in pSS pathogenesis and guide the development of more effective therapeutic strategies.

### 5.6 Chronic Inflammatory Diseases

Beyond systemic autoimmune disorders, BTKis are under growing investigation in chronic inflammatory diseases driven by Fc receptor‐mediated effector functions, eosinophil and mast cell activation, or persistent innate immune responses. In chronic spontaneous urticaria, remibrutinib has demonstrated rapid clinical improvement in wheal activity, pruritus, and overall disease burden in patients refractory to H1 antihistamines, with an acceptable safety profile [394, 395]. Atopic dermatitis, the most common form of eczema, has also been explored in this context. Although direct clinical data in atopic dermatitis remain limited, ibrutinib has been shown to inhibit IgE‐mediated basophil activation and reduce allergen‐induced responses of mast cells and basophils in adults with peanut or tree nut allergy [396]. The BTK pathway has also been implicated in airway inflammatory diseases, including asthma and chronic obstructive pulmonary disease (COPD), where persistent activation of innate immune cells and proinflammatory mediator release contribute to airway inflammation and remodeling. A phase II proof‐of‐concept study is currently evaluating rilzabrutinib in adults with moderate‐to‐severe asthma (NCT05104892) [397].

The therapeutic potential of BTK inhibition may thus extend beyond autoimmune diseases to a broader spectrum of chronic inflammatory and allergic conditions. Most indications, however, remain in early‐stage development, and additional clinical studies will be needed to establish long‐term efficacy, safety, and optimal patient selection in nonautoimmune inflammatory settings.

## 6 Conclusion and Future Prospects

This review has systematically covered the molecular biology, signaling networks, pharmacological evolution, and therapeutic applications of BTK inhibitors in human diseases. BTK is not merely a linear mediator of B‐cell receptor signaling but an interconnected immune signaling hub formed by the convergence of BCR, TLR, chemokine receptor, Fc receptor, and BAFF receptor pathways. Its core biological functions span receptor‐proximal kinase activation, B‐cell survival and differentiation, immune‐cell migration, innate immune modulation, and tumor–microenvironmental coordination. These interconnected mechanisms underpin the biological and translational rationale for BTK‐targeted therapy. The therapeutic paradigm of BTK inhibition has thus shifted from direct suppression of malignant B‐cell survival toward a broader framework integrating targeted inhibition, immune remodeling, and rational combination therapy. The progression from covalent to noncovalent inhibitors, dual‐target agents, and BTK degraders exemplifies this transition, providing diverse strategies to improve selectivity and overcome resistance.

### 6.1 Evolving Therapeutic Paradigm of BTK Inhibition

Over the past decade, BTK inhibitors have reshaped the therapeutic landscape of B‐cell malignancies, driving a paradigm shift from intensive chemoimmunotherapy toward targeted, oral, and chemotherapy‐free treatment strategies. This clinical transformation reflects the central role of BTK in oncogenic BCR signaling as well as its broader involvement in malignant B‐cell survival, proliferation, adhesion, migration, and tissue homing. By interrupting these interconnected processes, BTK inhibitors have established themselves as foundational agents across multiple B‐cell malignancy settings.

The therapeutic implications of BTK inhibition extend beyond direct tumor‐cell suppression. BTK inhibitors influence immune‐cell function, remodel tumor–microenvironmental interactions, and regulate cytokine‐mediated inflammatory responses, providing a biological basis for combination with cellular therapies and other immune‐based modalities. Notably, integrating BTK inhibitors with CAR‐T cell therapy offers new opportunities to modulate the immune microenvironment and potentially enhance therapeutic durability.

Beyond hematological oncology, BTK inhibitors have expanded into immune‐mediated diseases, exemplified by clinical advances and regulatory approvals in conditions such as ITP. This expansion reflects the expression and functional relevance of BTK in B cells and innate immune compartments, where it participates in autoantibody production, Fc receptor‐mediated effector responses, macrophage activation, mast‐cell signaling, and inflammatory modulation. BTK inhibition has thus evolved from a single‐agent strategy focused on malignant B‐cell signaling into a broader therapeutic platform integrating targeted inhibition, immune remodeling, and rational combination therapy.

### 6.2 Biological and Clinical Barriers to Durable Benefit

Despite these advances, durable disease control with BTK‐targeted therapy remains constrained by biological, clinical, and diagnostic challenges. Acquired resistance is the most prominent barrier. Initially characterized by C481 mutations that impair covalent BTK inhibitor binding, resistance is now recognized as a more heterogeneous process involving non‐C481 BTK mutations, PLCG2 alterations, alternative BCR signaling, bypass pathway activation, and kinase‐independent BTK scaffold functions. These mechanisms reveal that therapeutic failure is not merely a pharmacological consequence of reduced drug–target binding but also a product of clonal selection and adaptive signaling rewiring under continuous therapeutic pressure.

Long‐term disease control is further limited by cumulative toxicity and insufficient depth of remission. Although BTK inhibitors achieve high response rates in many B‐cell malignancies, monotherapy often fails to induce deep molecular remission such as MRD negativity, necessitating indefinite treatment. Continuous exposure may exacerbate cumulative toxicity and financial burden while increasing the risks of treatment discontinuation and clonal evolution.

### 6.3 Immunotherapy Combinations: Mechanistic Rationale and Clinical Uncertainty

BTK inhibition provides a compelling biological basis for immunotherapy‐based combinations. Beyond inhibiting oncogenic BCR signaling, BTK inhibition can reshape the immune landscape by enhancing T‐cell function, limiting tumor cell homing, and decreasing immunosuppressive cell populations [193]. These effects may alleviate T‐cell exhaustion, reduce immune checkpoint expression, and enhance antigen presentation, creating a more permissive environment for immune‐mediated tumor clearance.

The mechanistic foundation of these combinations, however, remains incompletely defined. Proposed mechanisms, including PI3K/AKT/mTOR signaling crosstalk, autophagy regulation, and reversal of T‐cell exhaustion, are still supported largely by associative or indirect evidence rather than definitive causal experiments. This limitation reflects the pleiotropic effects of BTK inhibition across malignant B cells, T cells, myeloid cells, and microenvironmental compartments, as well as the limited capacity of conventional syngeneic models to reproduce chronic antigen‐driven exhaustion in human disease [398]. Rigorous experimental validation is therefore needed to distinguish true mechanistic drivers from correlative observations [399]. More physiologically relevant models, including lineage‐specific BTK deletion systems, chronic viral infection models of exhaustion, and longitudinal single‐cell profiling of serial patient samples, may help bridge the translational gap between preclinical hypotheses and clinical validation [400].

Clinical evidence for BTK inhibitor‐based immunotherapy combinations also remains heterogeneous. Much of the available evidence derives from retrospective analyses, small single‐arm phase I/II studies, or institutional cohorts with heterogeneous CAR‐T constructs and supportive care protocols. BTK inhibitors have also been administered at different time points relative to CAR‐T therapy, including during bridging, peri‐infusion, or postinfusion maintenance, yet the impact of treatment timing on CAR‐T expansion, persistence, functional fitness, and immune‐related toxicity remains insufficiently characterized. Prospective trials with standardized CAR‐T platforms and integrated correlative studies are therefore required to define optimal sequencing and duration and to evaluate efficacy and safety.

Similar considerations apply to combinations with immune checkpoint inhibitors, CD20×CD3 bispecific antibodies, and other immune‐based approaches. While early‐phase studies suggest feasibility and potential additive or synergistic activity, durable benefit, optimal patient selection, and long‐term safety remain to be established. Future investigations should move beyond exploratory combination design and incorporate translational endpoints, including immune profiling, MRD assessment, NGS‐based resistance monitoring, and functional immune assays, to identify patients most likely to benefit from specific BTK inhibitor‐based combinations.

### 6.4 Precision‐Guided Strategies for Next‐Generation BTK‐Targeted Therapy

Emerging BTK‐targeted agents offer new opportunities to overcome resistance and improve clinical outcomes. Noncovalent inhibitors and dual‐mode compounds such as LP‐168, which bind BTK in mutation‐dependent contexts, increase therapeutic flexibility in resistant disease. BTK degraders, including NX‐2127, eliminate BTK via the ubiquitin–proteasome system and may bypass kinase‐impaired mutations [136], while dual LYN/BTK inhibitors such as birelentinib (DZD8586) suppress both BTK‐dependent and alternative BCR signaling [134]. These agents overcome acquired resistance through distinct mechanisms of action and may serve as a more adaptable backbone for rational immune‐based combinations, including CAR‐T therapy, bispecific antibodies, BCL‐2 inhibition, and checkpoint blockade, enabling systematic evaluation of optimal combination partners and sequencing strategies, particularly within time‐limited or MRD‐guided frameworks.

Innovation alone, however, does not eliminate biological constraints. Large‐molecule degraders face pharmacokinetic limitations [401], safety signals such as arrhythmia have emerged [402], and resistance mutations (e.g., A428D) highlight ongoing evolutionary adaptation under therapeutic pressure [403]. The clinical value of next‐generation BTK‐targeted agents will therefore depend not only on potency or novel mechanisms of action but also on rational sequencing, safety optimization, and dynamic treatment monitoring.

Looking ahead, several directions hold promise. First, molecular resistance testing and next‐generation BTK inhibitor development should be integrated into BTK‐targeted therapeutic strategies to clarify clonal evolution under continuous treatment pressure. Targeted next‐generation sequencing (NGS)‐based assays, complemented by cfDNA analysis and single‐cell genotyping where appropriate, may detect emerging resistant subclones before overt clinical relapse, particularly alterations involving BTK, PLCG2, BCL2, and other resistance‐associated genes, shifting resistance monitoring from retrospective interpretation toward prospective treatment switching, combination intensification, and therapeutic sequencing [404]. Concurrently, noncovalent inhibitors, dual‐target or dual‐mode inhibitors, and BTK degraders should be developed with consideration of defined resistance mechanisms, kinase‐independent BTK functions, pharmacokinetic properties, and safety profiles. Second, response‐adapted trials combining BTK inhibitors with CAR‐T cells, bispecific antibodies, ICIs, or BCL2 inhibition should define the optimal timing, duration, immune context, and toxicity profile of these strategies. Third, multi‐omics technologies, including single‐cell sequencing, spatial transcriptomics, proteomics, and TCR/BCR repertoire profiling, may delineate BTK‐dependent immune networks across malignant, autoimmune, inflammatory, and selected solid tumor settings. By defining disease‐specific BTK activity in B cells, myeloid cells, mast cells, stromal compartments, and tumor‐associated immune niches, these approaches may distinguish causal mechanisms from correlative observations, identify biomarkers of response, resistance, and toxicity, and guide the rational expansion of BTK inhibitors beyond hematologic malignancies. This is particularly important for benign immune‐mediated diseases, where future development should be guided by patient selection, tissue distribution, long‐term safety thresholds, and disease‐specific mechanisms rather than direct extrapolation from oncology experience.

Ultimately, BTK‐targeted therapy is entering a stage in which therapeutic success will depend less on expanding indications alone than on matching the right inhibitor, combination partner, treatment duration, and monitoring strategy to the biological context of each disease. By integrating next‐generation BTK‐targeted agents, molecular resistance testing, immune profiling, and multi‐omics‐guided biomarker discovery, future treatment models may move from continuous empirical suppression toward mechanism‐informed, response‐adapted, and potentially time‐limited intervention. Such an approach may improve therapeutic durability, reduce unnecessary toxicity, and extend the clinical value of BTK inhibition across malignant and immune‐mediated diseases.

## Author Contributions

M.S. drafted the manuscript and created the figures and tables. J.W. and D.J. reviewed, revised, and contributed to the manuscript and the figures and tables. All authors have read and approved the final manuscript.

## Funding

This work was supported by grants from the National High Technology Research and Development Program of China (Grant 2021YFA1101500, to Professor Jia Wei) and the National Natural Science Foundation of China (Grant 82070217, to Professor Jia Wei).

## Ethics Statement

The authors have nothing to report.

## Conflicts of Interest

The authors declare no conflicts of interest.

## Data Availability

This article did not include any new data production or analysis. Therefore, data sharing does not apply.
